# The beneficial effects of *Lactobacillus reuteri* ADR-1 or ADR-3 consumption on type 2 diabetes mellitus: a randomized, double-blinded, placebo-controlled trial

**DOI:** 10.1038/s41598-018-35014-1

**Published:** 2018-11-14

**Authors:** Ming-Chia Hsieh, Wan-Hua Tsai, Yu-Pang Jheng, Shih-Li Su, Shu-Yi Wang, Chi-Chen Lin, Yi-Hsing Chen, Wen-Wei Chang

**Affiliations:** 1 Graduate Institute of Integrative Medicine, China Medical University, Taichung, 40402 Taiwan; 2 Division of Endocrinology and Metabolism, Department of Internal Medicine, Changhua Christian Hospital, Changhua, 500 Taiwan; 3 Research and Development Department, GenMont Biotech Incorporation, Tainan, 74144 Taiwan; 4 Department of Life Sciences, Institute of Biomedical Science, National Chung Hsing University, Taichung, 40249 Taiwan; 5 Department of Biomedical Sciences, Chung Shan Medical University, Taichung, 40201 Taiwan; 6 Department of Medical Research, Chung Shan Medical University Hospital, Taichung, 40201 Taiwan

## Abstract

Probiotics have been reported to ameliorate symptoms of type 2 diabetes mellitus (T2DM) in animal models and human studies. We previously demonstrated that oral administration of *Lactobacillus reuteri* ADR-3 reduced insulin resistance in high-fructose-fed (HFD) rats. In the present study, we first identified another *L*. *reuteri* strain, ADR-1, which displayed anti-diabetes activity that reduced the levels of serum HbA1c and cholesterol and that increased antioxidant proteins in HFD rats. We further performed a randomized, double-blinded, placebo-controlled trial with a total of 68 T2DM patients to examine the beneficial effects of oral consumption of *L*. *reuteri* strains ADR-1 and ADR-3 and to investigate the associated changes in intestinal flora using a quantitative PCR method to analyze 16 S rRNA in fecal specimens. Significant reductions in HbA1c and serum cholesterol were observed in participants in the live ADR-1 consumption group (n = 22) after 3 months of intake when compared with those in the placebo group (n = 22). Although there was no significant difference in the HbA1c serum level among participants who consumed heat-killed ADR-3 (n = 24), the systolic blood pressure and mean blood pressure were significantly decreased after 6 months of intake. There was no obvious change in serum inflammatory cytokines or antioxidant proteins in participants after intaking ADR-1 or ADR-3, except for a reduction in IL-1β in the ADR-3 consumption group after 6 months of intake. With the analysis of fecal microflora, we found that *L*. *reuteri* or *Bifidobacterium* spp. were significantly increased in the ADR-1 and ADR-3 consumption groups, respectively, after 6 months of intake. Interestingly, a significant reduction in HbA1c was observed in the ADR-1 and ADR-3 consumption participants who displayed at least an 8-fold increase in fecal *L*. *reuteri*. We also observed that there was a significantly positive correlation between *Bifidobacterium* spp. and *Lactobacillus* spp. in participants with increased levels of fecal *L*. *reuteri*. In the ADR-1 intake group, the fecal *Lactobacillus* spp. level displayed a positive correlation with *Bifidobacterium* spp. but was negatively correlated with *Bacteroidetes*. The total level of fecal *L*. *reuteri* in participants in the ADR-3 consumption group was positively correlated with *Firmicutes*. In conclusion, *L*. *reuteri* strains ADR-1 and ADR-3 have beneficial effects on T2DM patients, and the consumption of different strains of *L*. *reuteri* may influence changes in intestinal flora, which may lead to different outcomes after probiotic intake.

## Introduction

Type 2 diabetes mellitus (T2DM) is a chronic metabolic disorder with peripheral insulin resistance and is the most common form of diabetes, with an almost 2-fold increased incidence in the past two decades^1^. The complications of T2DM include hypertension, nephropathy, neuropathy, skin infections and a high risk of blindness^2^. There were 1.6 million deaths directly caused by diabetes, and the World Health Organization predicts that diabetes will be the seventh leading cause of death in 2030^1^. Consequently, there is an urgent need to develop functional health care products for regulating blood sugar.

Probiotics were defined by the Joint FAO/WHO Expert Consultation in 2002 as living microorganisms with health benefits to humans when administered in adequate amounts^3^. It has been demonstrated that probiotics have beneficial effects on several disorders; these beneficial effects include a reduction in allergic symptoms^4,5^, a decrease in serum cholesterol levels^6^, the prevention of obesity^7^ and improvement of the digestive system^8,9^. In T2DM studies, there have been several reports demonstrating that probiotics have beneficial effects on controlling blood sugar. For example, in pregnant women, daily consumption of probiotic yogurt consisting of two strains of *Lactobacilli* and *Bifidobacteria* for 9 weeks helped to maintain serum insulin levels^10^. In another study, after receiving *Lactobacillus gasseri* BNR17, db/db mice displayed an improvement in glucose sensitivity and amelioration of diabetic symptoms such as polydipsia, hyperplasia and polyuria^11^. We previously demonstrated that oral administration of *Lactobacillus reuteri* GNML-263 (ADR-3) reduced insulin resistance and hepatic steatosis formation in high-fructose-fed (HFD) rats, an animal model of T2DM^12^. This finding suggests that ADR-3 has the potential to be developed as a health care product for controlling T2DM complications.

In the present study, we further selected another *L*. *reuteri* strain, ADR-1, that has been demonstrated to have beneficial effects on HDF rats, such as reductions in serum HbA1c and liver injury after oral gavage for 14 weeks. In a double-blinded, randomized, placebo-controlled trial with a total of 68 patients who consumed live ADR-1 or heat-killed ADR-3 daily for 6 months, the results indicated that oral administration of ADR-1 significantly reduced HbA1c, whereas consumption of ADR-3 significantly decreased the systolic blood pressure (SBP) and mean blood pressure (MBP) after 6 months. The analysis of fecal microflora after consumption of ADR-1 or ADR-3 indicated that the different outcomes of ADR-1 or ADR-3 consumption in T2DM patients may be associated with changes in intestinal flora. Our data suggest that *L*. *reuteri* strains ADR-1 and ADR-3 have the potential to be developed as health care products for the management of T2DM.

## Materials and Methods

### Examining the beneficial effects of ADR-1 on a T2DM rat model

Male Sprague-Dawley rats were purchased from the National Laboratory Animal Center (Taipei, Taiwan) and divided into three groups: (1) the control group (n = 8), which consumed a standard 65% cornstarch diet (PMI Nutrition International, Brentwood, MO, USA); (2) the high-fructose-fed (HFD) group (n = 8), which consumed a 65% fructose diet (Harlan Laboratories, Inc., USA); and (3) the HFD with oral gavage of *L*. *reuteri* ADR-1 group (n = 8), which was simultaneously fed a 65% fructose diet and ADR-1 (2 × 10^9^ CFU/rat) every day for 14 weeks. Blood samples were collected, and HbA1c, low-density lipoprotein-cholesterol (LDL), high- density lipoprotein-cholesterol (HDL), cholesterol (CHOL), triglycerides (TG), aspartate aminotransferase (AST), and alanine transaminase (ALT) were measured by the National Laboratory Animal Center Biological Laboratory (Taipei, Taiwan). A glutathione peroxidase assay kit (Cat# 703102, CAYMAN, USA) and superoxide dismutase assay kit (Cat# 706002, CAYMAN, USA) were used in this study. The animal experiments were approved by the Institutional Animal Care and Use Committee of GenMont Biotech Inc. (Tainan, Taiwan, IACUC No. AB10009-2) and performed in accordance with the relevant guidelines and regulations.

### The recruitment of participants

A total of 86 people with T2DM were recruited for eligibility assessment through local advertisements, and 12 people were excluded (Fig. 2A). The recruitment criteria were as follows: T2DM diagnosis more than 6 months ago, age ranging from 25–70 years, a body-mass index (BMI) of more than 18.5; and an HbA1c level of 7% to 10%. The exclusion criteria were as follows: pregnancy; the presence of other diseases, including cancers (with the exception of well-controlled benign tumors), kidney failure/dialysis, heart diseases, stroke, or autoimmune diseases; the use of medications, including anti-diabetes drugs, and antibiotics, or other probiotic products <4 weeks before randomization; an AST/ALT level >3-fold the normal range; participation in other clinical trials; and the presence of other medical conditions that might jeopardize compliance with the protocol (e.g., malabsorption syndrome or an inability to take orally administered drugs). All the eligible participants were given verbal and written information about the study before being asked to sign a written consent form.

### Study design

A 9-month double-blinded, randomized, placebo-controlled study was performed. The study was approved by the Ethics Committee of Changhua Christian Hospital with approval No. 140703 and performed in accordance with the relevant guidelines and regulations. The trial protocol is available in the supplementary materials. This study was registered at Clinical Trials.gov with the identifier NCT02274272 on Oct 24, 2014. At the first visit, participants underwent a baseline examination, including the recording of demographic characteristics and collection of fasting blood, urine, and stool samples. A total of 74 participants were double-blinded and randomized into 3 groups: live *L*. *reuteri* ADR-1 (n = 25), heat-killed *L*. *reuteri* ADR-3 (n = 25), and placebo (n = 24). In the ADR-1 and ADR-3 consumption groups, participants were orally administered 4 × 10^9^ CFU of ADR-1 or 2 × 10^10^ cells of ADR-3 every day, respectively. ADR-1 and ADR-3 were given as capsules (2 × 10^9^ CFU/capsule or 1 × 10^10^ cells/capsule for ADR-1 or ADR-3, respectively), which were provided by GenMont Biotech Inc. (Taiwan). The accuracy of *L*. *reuteri* ADR-1 (China Center for Type Culture Collection [CCTCC] number: M207154) and *L*. *reuteri* ADR-3 (CCTCC number: M209263) was confirmed by 16S rRNA sequencing and random amplified polymorphic DNA (RAPD) methods (Fig. S1). All participants were called back for the collection of fasting blood and urine samples at visit 2 (3 months), visit 3 (6 months), and visit 4 (orally administered probiotic washed out for 3 months). The poststudy stool samples of participants were collected at visit 3.

### Laboratory and cytokine measurements

Fasting serum samples were used for biochemical analyses, including TG, CHOL, LDL, HDL, CHOL, AST, ALT, insulin, C-peptide, free fatty acid, BUN, CRE, eGFR, HbA1c, and glucose analyses, at Union Clinical Laboratory (Taiwan, TAF No. L1447-150325/CAP No. 6979606). The cytokine profiles were determined by enzyme-linked immunosorbent assay (ELISA) kits and performed at the Department of Life Sciences (Institute of Biomedical Science, National Chung Hsing University, Taichung, Taiwan). The ELISA kits included human IL-6 (Cat# 900-K16, PeproTech, USA), human IL-10 (Cat# 900-K21, PeproTech), human IL-17A (Cat# 900-K84, PeproTech), human TNF-α (Cat# 50-114-2609, eBioscience, USA), and human IL-1β (Cat# 437005, Biolegend, USA). A glutathione peroxidase assay kit (Cat# 703102, CAYMAN, USA) and superoxide dismutase assay kit (Cat# 706002, CAYMAN) were used in this study according to the manufacturers’ standard protocol.

### Analysis of fecal microbiota compositions

All 180–220 mg stool samples were collected in RNAlater^TM^ (Thermo Fisher Scientific, Waltham, MA, USA)-containing tubes with autoclaved glass beads, and DNA was extracted with a QIAamp DNA Stool Mini Kit (QIAGEN, Hilden, Germany) after homogenization by MagNA Lyser (Roche Diagnostics Corporation, IN, USA). Furthermore, 2 ng of extracted DNA was used for analysis of the fecal microbiota composition by quantitative PCR, which was performed with a commercial Rotor-Gene^TM^ SYBR Green PCR Kit (QIAGEN, Germany) and measured on a Rotor-Gene Q system (QIAGEN, Germany). The primer sets are listed in Table S1. The changes in specific bacteria were calculated by the 2^−ΔΔCt^ method, in which ΔΔCt was [Ct(target bacteria at 6 months)-Ct(total bacteria at 6 months)]-[Ct(target bacteria at the starting time point)-Ct(total bacteria at the starting time point)].

### Statistical analysis

All statistical analyses were performed by a private company (Bestat Pharma services corp., Taiwan) with SAS software version 9.4 (SAS Institute, Cary, NC). Baseline information was analyzed using the chi-square test or two-sample *t*-test. Medical histories, medication for diabetes, and hypertension in the different groups were analyzed using Fisher’s exact test. Comparisons of the change in results between the probiotic and placebo groups were analyzed using the two-sample *t*-test. Correlations between the variables were computed by Spearman’s rho correlation provided by PASW Statistics 18 Software (SPSS Inc., Chicago, IL, USA). A *p* value less than 0.05 was considered statistically significant.

## Results

### *L*. *reuteri* strain ADR-1 displayed beneficial effects in a T2DM rat model

**Figure 1:**
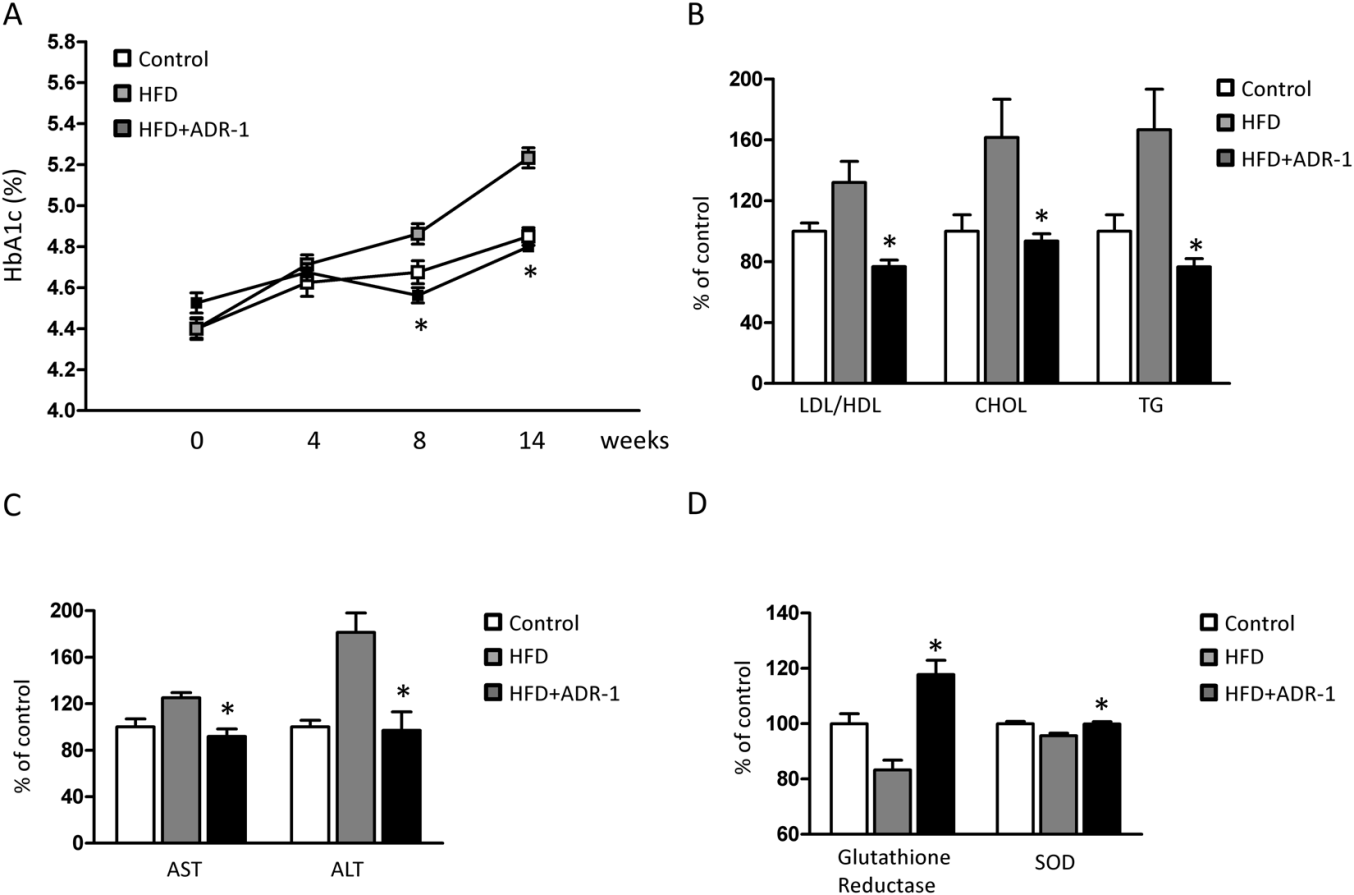
The beneficial effects of ADR-1 in a T2DM rat model. Male Sprague-Dawley rats were divided into three groups as control (normal diet), HFD (high fructose diet with oral gavage of water), and HDF + ADR-1 (high fructose diet with oral gavage of ADR-1 as 2 × 10^9^ cfu/rat/day). (**A**) The changes of HbA1c among groups were determined. Data was shown as mean±SD. *p < 0.05, comparing with HFD group. (**B**) The effects of ADR-1 in blood lipids of HFD rats. (**C**) The effect of ADR-1 in HDF induced liver injury was determined by serum AST/ALT activity. (**D**) The changes of hepatic antioxidant enzymes, glutathione reductase and superoxide dismutase (SOD) were determined. The value of control group was calculated as 100%. Data was shown as mean ± SD. *p < 0.05, comparing with HFD group.

We previously reported that oral administration of live *L*. *reuteri* ADR-3 improves insulin resistance and ameliorates hepatic steatosis in HFD rats^12^. In addition to *L*. *reuteri* ADR-3, we further examined if another *L*. *reuteri* strain, ADR-1, displays similar effects. With daily oral gavage of live *L*. *reuteri* ADR-1 at 2 × 10^9^ CFU/rat, the serum HbA1c level in HFD rats was significantly reduced at the 8^th^ week and the 14^th^ week in comparison with that in the HFD only group (Fig. 1A). The levels of CHOL and TG and the ratio of LDL/HDL were significantly reduced in HFD rats receiving ADR-1 orally (Fig. 1B). The decreased serum levels of aspartate transaminase (AST) and alanine transaminase (ALT) demonstrated that liver injury in HFD rats was ameliorated by oral gavage of ADR-1 (Fig. 1C). Reductions in serum antioxidant proteins such as glutathione reductase (GPX) and superoxide dismutase (SOD) were also observed in HFD rats with oral administration of ADR-1 (Fig. 1D). These data indicate that *L*. *reuteri* ADR-1 has clinical potential for the management of T2DM.

### A double-blinded, randomized, placebo-controlled trial of live *L*. *reuteri* ADR-1 and heat-killed *L*. *reuteri* ADR-3 in T2DM patients

**Figure 2:**
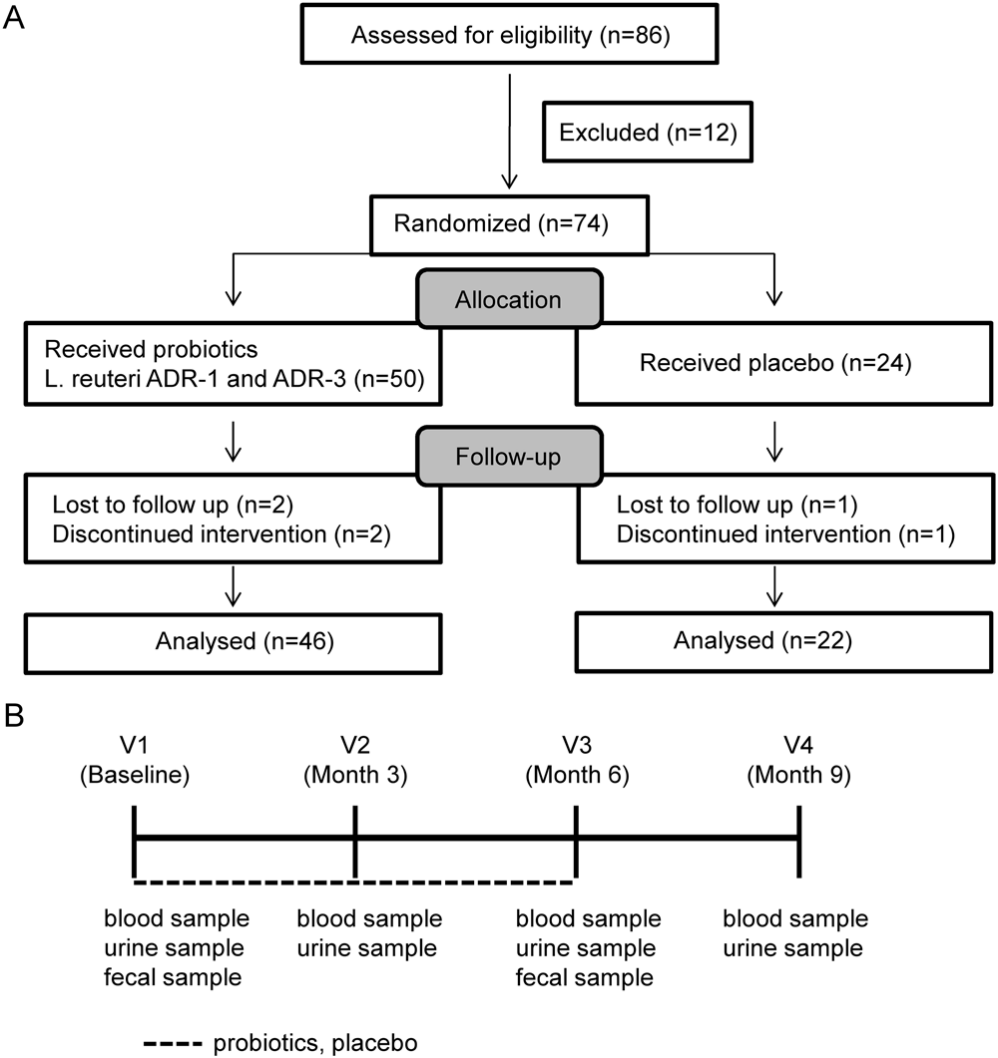
Recruitment of participants and the study design of a double blinded, randomized, and placebo controlled trial. (**A**) A total of 86 T2DM patients came for assessment of eligibility and excluded for 12 people. A total of 74 eligible participants was randomized divided into live *L*. *reuteri* ADR-1, heat-killed *L*. *reuteri* ADR-3, or placebo group. After exclusion for participates with loss of follow up or discontinued intervention, there was 46 or 22 analyzed participates for probiotics consumption or placebo group, respectively. (**B**) The probiotics consumption was performed a total of 6 months and wash out the probiotics for further 3month. All participates were requested to collect fasting blood and urine samples at 4 visits (V1: starting point, V2: 3 months after consumption, V3: 6 months after consumption, and V4: 3 months after stopping consumption) as indicated. The fecal samples were collected at V1 and V3.

We previously demonstrated that heat-killed ADR-3 displayed a similar effect to live ADR-3 in the improvement of metabolic function in high-fat diet induced obese rats^13^. Heat-killed probiotics have an advantage when used in subjects with immunodeficiency^14^. To understand if live ADR-1 or heat-killed ADR-3 has beneficial effects on T2DM patients, we conducted a 9-month, double-blinded, randomized, placebo-controlled trial (Clinical Trial Number at ClinicalTrials.gov: NCT02274272) as described in the Materials and Methods section. The study design is shown in Fig. 2, and the times of visits are indicated as V1 (baseline before trial), V2 (3 months after consumption), V3 (6 months after consumption), and V4 (3 months after stopping consumption). At V1, a total of 74 participants underwent a baseline examination to collect demographic data and fasting blood, urine and fecal samples and to measure blood pressure. A total of 6 participants discontinued the intervention or were lost to follow-up, while the other 68 participants completed the trial. However, one participant lost a fecal sample. The baseline information of the participants is listed in Table S1. There were no significant differences in the gender ratio, body weight, blood pressure, liver function (AST/ALT level), the HbA1c level (Table S2) and medication history (Table S3) among the groups at the beginning of the trial.

### The effects of live *L*. *reuteri* ADR-1 or heat-killed *L*. *reuteri* ADR-3 consumption on HbA1c and other metabolic markers

**Figure 3:**
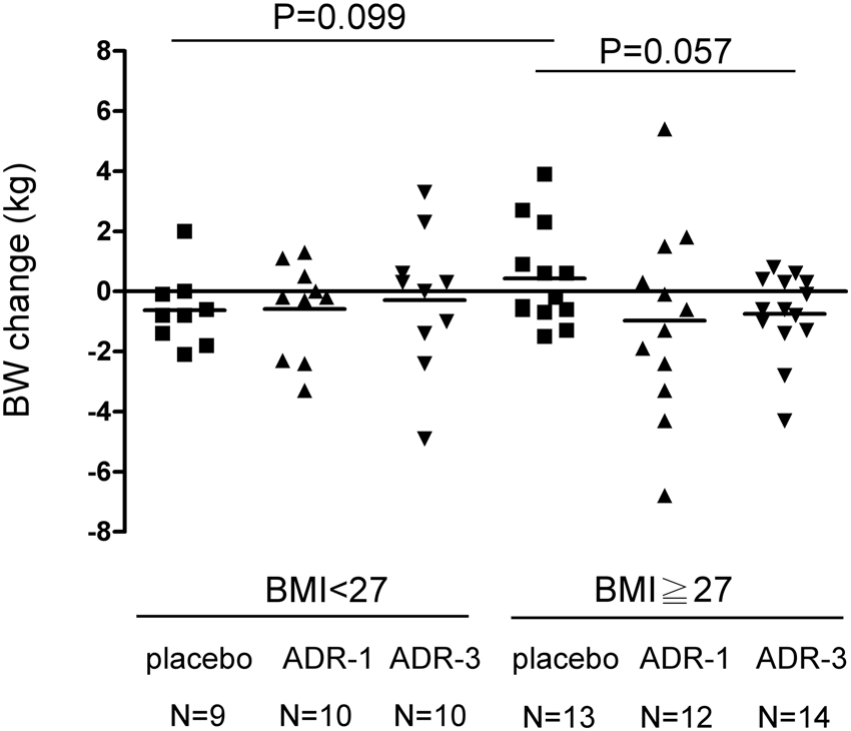
The correlation between probiotics consumption and body weight control in T2DM patients. Participants were divided into two groups according to BMI (<27 or ≥27) and the changes of body weight (BW) among groups were analyzed by two sample *t*-test.

The HbA1c net change level in the *L*. *reuteri* ADR-1 consumption group was significantly reduced at V2, V3, and V4 (Table 1 and Fig. S2A, *p* = 0.0321 for V2, *p* = 0.0212 for V3, and *p* = 0.0285 for V4). These results indicated that participants who consumed *L*. *reuteri* ADR-1 for 6 months could maintain a stable HbA1c level for 3 months after stopping intake. For the comparison of HbA1c levels among the three groups at different times throughout the intervention, only participants from the live *L*. *reuteri* ADR-1 intake group displayed a decreased trend in the HbA1c level, but a significant difference was observed at only V4 (Fig. S2B, *p* = 0.0494). We did not observe any significant net change in HbA1c at any time points in the heat-killed *L*. *reuteri* ADR-3 consumption group (Table 1). The net changes in insulin, HOMA-IR, and fasting blood glucose (glucose-AC) among groups were not significant in comparison with those in the placebo group (Table 1). For other metabolic markers, we observed a trend in decreasing blood lipids in the ADR-1 consumption group. Reductions in LDL, cholesterol, and free fatty acids were observed in the ADR-1 consumption group at V2, although only cholesterol was significantly reduced (Table 1, *p* = 0.0467). On the other hand, there was no significant change in blood lipids for the ADR-3 consumption group, but significant reductions in systolic blood pressure (SBP) and mean blood pressure (MBP) were observed (Table 1, *p* = 0.0248 for SBP and *p* = 0.0254 for mean pressure). We also observed that bodyweight control in participants with obesity (body mass index greater than 27) was better in the ADR-3 consumption group (Fig. 3).

**Table 1:** Clinical-biochemical data in participates after *L*. *reuteri* consumption.

		placebo^a^	ADR-1^a^	P-value^b^	ADR-3^a^	P-value^b^
		N = 22	N = 22		N = 24	
HbA1c (%)	3M-0M	0.22 ± 0.93	−0.35 ± 0.74	0.0321	0.07 ± 0.67	0.5509
	6M-0M	0.22 ± 0.87	−0.39 ± 0.80	0.0212	0.24 ± 0.93	0.9427
Insulin (mU/L)	3M-0M	8.00 ± 36.76	−4.15 ± 17.86	0.1731	4.45 ± 21.24	0.6944
	6M-0M	1.70 ± 9.08	−3.14 ± 11.46	0.1282	16.25 ± 43.90	0.1249
HOMA-IR	3M-0M	0.14 ± 5.57	−1.83 ± 8.01	0.3500	0.03 ± 9.27	0.9605
	6M-0M	−0.12 ± 4.16	−0.91 ± 5.82	0.6082	6.57 ± 19.17	0.1079
Glucose AC (mg/dl)	3M-0M	−9.36 ± 45.86	−1.36 ± 22.53	0.4683	−11.20 ± 53.96	0.9038
	6M-0M	−10.40 ± 53.00	−0.32 ± 31.92	0.4515	−9.38 ± 58.45	0.9525
LDL (mg/dl)	3M-0M	3.73 ± 24.30	−8.18 ± 17.47	0.0690	8.58 ± 14.82	0.4239
	6M-0M	−5.09 ± 25.13	−4.50 ± 16.29	0.9267	1.79 ± 24.32	0.3505
HDL (mg/dl)	3M-0M	1.18 ± 7.58	−1.45 ± 9.50	0.3145	−0.25 ± 5.19	0.4552
	6M-0M	−0.91 ± 9.73	0.32 ± 6.61	0.6272	−1.67 ± 5.45	0.7497
TG (mg/dl)	3M-0M	2.68 ± 96.38	−12.30 ± 81.99	0.5823	8.71 ± 55.45	0.7989
	6M-0M	22.18 ± 92.62	−22.70 ± 68.88	0.0754	61.63 ± 213.90	0.4165
CHOL (mg/dl)	3M-0M	4.77 ± 23.97	−9.86 ± 23.40	0.0467	9.04 ± 19.46	0.5091
	6M-0M	−1.55 ± 25.34	−4.45 ± 20.94	0.6802	8.17 ± 40.99	0.3355
FFA (mmol/L)	3M-0M	−0.04 ± 0.50	−0.28 ± 0.41	0.0935	−0.13 ± 0.51	0.5287
	6M-0M	0.15 ± 0.61	−0.15 ± 0.46	0.0712	0.02 ± 0.56	0.4540
SBP (mmHg)	3M-0M	−0.09 ± 15.06	−4.55 ± 14.00	0.3155	−6.33 ± 14.96	0.1659
	6M-0M	1.95 ± 13.93	−2.82 ± 11.03	0.2146	−7.54 ± 13.77	0.0248
DBP (mmHg)	3M-0M	−0.59 ± 9.59	−2.77 ± 9.55	0.4537	−3.33 ± 5.79	0.2537
	6M-0M	0.36 ± 8.28	−0.91 ± 6.71	0.5783	−3.17 ± 5.45	0.0921
pulse pressure (mmHg)	3M-0M	0.5 ± 11.08	−1.77 ± 9.23	0.4641	−3 ± 11.71	0.3034
	6M-0M	1.59 ± 11.68	−1.91 ± 8.69	0.2664	−4.38 ± 12.54	0.1019
mean pressure (mmHg)	3M-0M	−0.42 ± 10.47	−3.36 ± 10.35	0.3545	−4.33 ± 8.16	0.1682
	6M-0M	0.89 ± 8.95	−1.55 ± 7.34	0.3285	−4.63 ± 6.94	0.0254

Abbreviations: HOMA-IR, homeostatic model assessment-insulin resistance; LDL, low-density lipoprotein; HDL, high-density lipoprotein; TG, triglycerides; CHOL, cholesterol; FFA, free fatty acid; SBP, systolic blood pressure; DBP, diastolic blood pressure; PP, pulse pressure; MBP, mean blood pressure.

^a^Data were calculated by the value of later time point (3 month (3 M) or 6 month (6 M)) minus to starting point (0 M) and presented as mean ± SD.

^b^Two sample *t*-test analysis was used to compare mean values between placebo and ADR-1 or ADR-3 groups.

### The effects of *L*. *reuteri* ADR-1 or ADR-3 consumption on inflammatory cytokines or antioxidant proteins

Epidemiologic studies have demonstrated a strong association between inflammatory and oxidative conditions and the occurrence of complications in T2DM patients^15,16^. Therefore, we next examined the changes in inflammatory cytokines and antioxidant proteins in the ADR-1 or ADR-3 consumption group in comparison with those in the placebo group. Among the inflammatory cytokines analyzed, only IL-1β displayed a significant reduction in the ADR-3 consumption group at V3 (Table 2, *p* = 0.0181). The SOD expression tended to be increased in the ADR-1 consumption group at V3 but did not reach a significant difference (Table 2, *p* = 0.0833).

**Table 2:** The changes of inflammatory cytokines and anti-oxidant enzymes in participates after *L*.*reuteri* consumption.

Cytokine/anti-oxidant enzyme	Time point	placebo^a^	ADR-1^a^	P value^b^	ADR-3^a^	P value^b^
		N = 22	N = 22		N = 24	
C-peptide (ng/ml)	3M-0M	−0.16 ± 0.49	0.03 ± 0.89	0.3773	−0.22 ± 0.62	0.7550
	6M-0M	0.05 ± 0.45	−0.03 ± 0.46	0.5447	−0.13 ± 0.63	0.2665
CRP (mg/dl)	3M-0M	−0.04 ± 0.18	0.04 ± 0.27	0.2697	0.06 ± 0.25	0.1282
	6M-0M	0.08 ± 0.28	0.03 ± 0.19	0.5483	0.05 ± 0.24	0.7636
IL-6 (ng/ml)	3M-0M	0.51 ± 1.30	−0.07 ± 2.34	0.3121	0.61 ± 1.69	0.8382
	6M-0M	0.90 ± 1.80	0.95 ± 2.65	0.9461	1.55 ± 2.41	0.3189
IL-10 (ng/ml)	3M-0M	1.43 ± 4.04	0.26 ± 1.63	0.2313	3.33 ± 12.16	0.4752
	6M-0M	1.04 ± 2.41	1.48 ± 3.09	0.6109	2.05 ± 3.25	0.2469
IL-17 (ng/ml)	3M-0M	−0.38 ± 2.54	0.60 ± 3.04	0.2630	−0.16 ± 2.32	0.7663
	6M-0M	−0.35 ± 3.63	0.66 ± 3.24	0.3464	0.47 ± 2.91	0.4097
TNF-α (pg/ml)	3M-0M	−20.00 ± 58.19	−3.36 ± 72.09	0.4265	−10.60 ± 50.26	0.5799
	6M-0M	−3.07 ± 72.22	−32.00 ± 81.24	0.2317	12.81 ± 86.00	0.5191
IL-1β (pg/ml)	3M-0M	0.00 ± 0.88	−0.69 ± 1.96	0.1604	−0.82 ± 2.69	0.1823
	6M-0M	0.21 ± 1.52	−0.72 ± 1.94	0.1027	−1.43 ± 2.70	0.0181
SOD (U/ml)	3M-0M	0.27 ± 0.82	0.85 ± 1.32	0.1338	−0.24 ± 1.00	0.1001
	6M-0M	0.39 ± 0.82	0.93 ± 0.95	0.0833	0.04 ± 1.31	0.3540
GPX (U/ml)	3M-0M	−0.62 ± 4.77	−1.57 ± 2.89	0.4908	−0.16 ± 4.66	0.7681
	6M-0M	−0.40 ± 3.79	−0.34 ± 3.24	0.9629	−0.27 ± 4.36	0.9256

Abbreviations: CRP, C-reactive protein; IL, interleukin; TNF, tumor necrosis factor; SOD, superoxide dismutase; GPX, glutathione peroxidase.

^a^Data were calculated by the value of later time point (3 month (3 M) or 6 month (6 M)) minus to starting point (0 M) and presented as mean ± SD.

^b^Two sample *t*-test analysis was used to compare mean values between placebo and ADR-1 or ADR-3 groups.

### The changes in intestinal flora in T2DM participants in the ADR-1 and ADR-3 consumption groups

**Figure 4:**
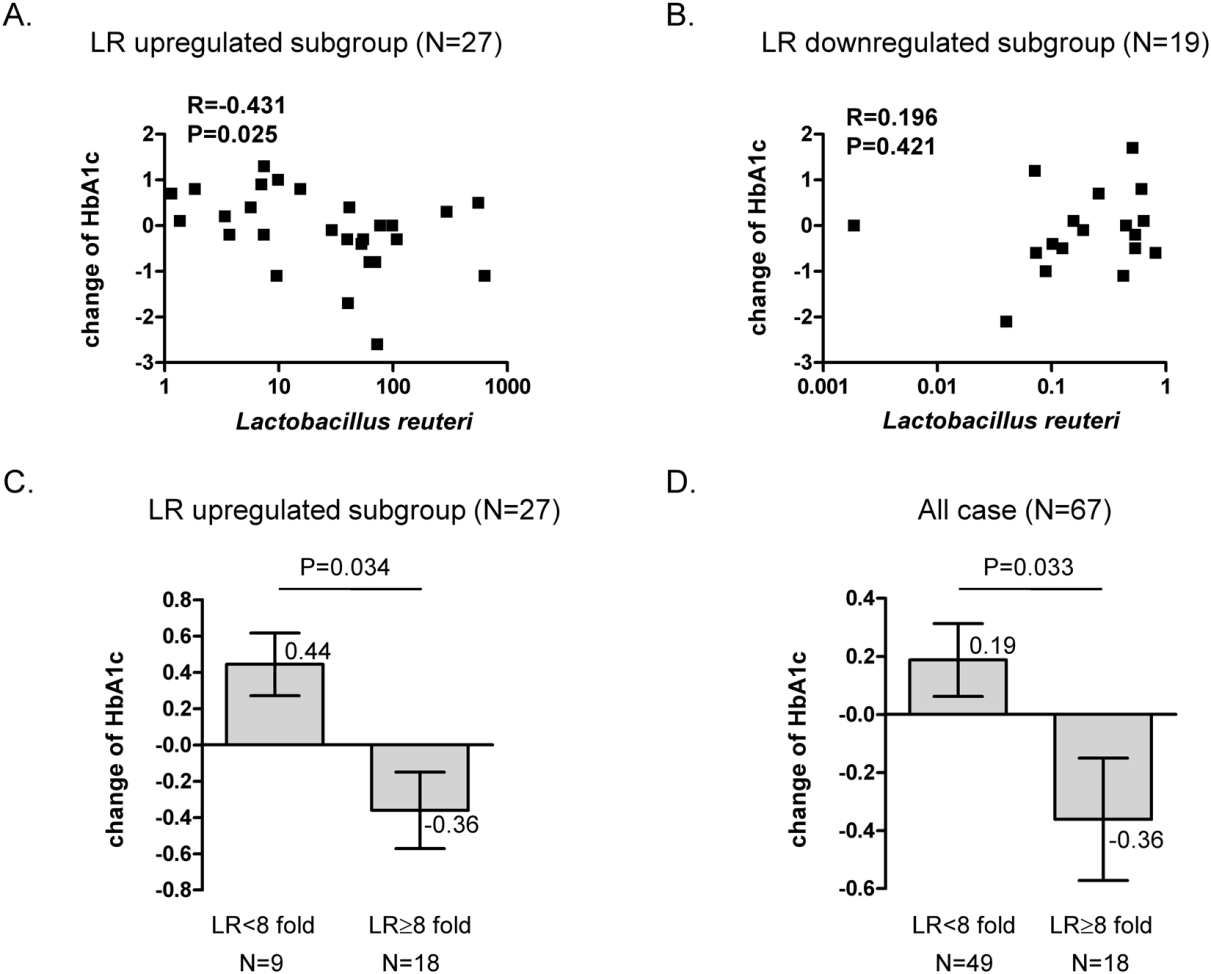
The association between fecal *L*. *reuteri* level and the changes of HbA1c. The correlation between *L*. *reuteri* level and the change of HbA1c in participants with *L*. *reuteri* level up-regulated group (LR change>1) (**A**) or down-regulated group (LR change≤1) (**B**). The statistical analysis was calculated with Spearman’s rho correlation. (**C**,**D**) Analysis of HbA1c changes between participants with different level of upregulated *L*. *reuteri* (**C**), n = 27) or all cases involving in this trial (**D**), n = 67).

**Figure 5:**
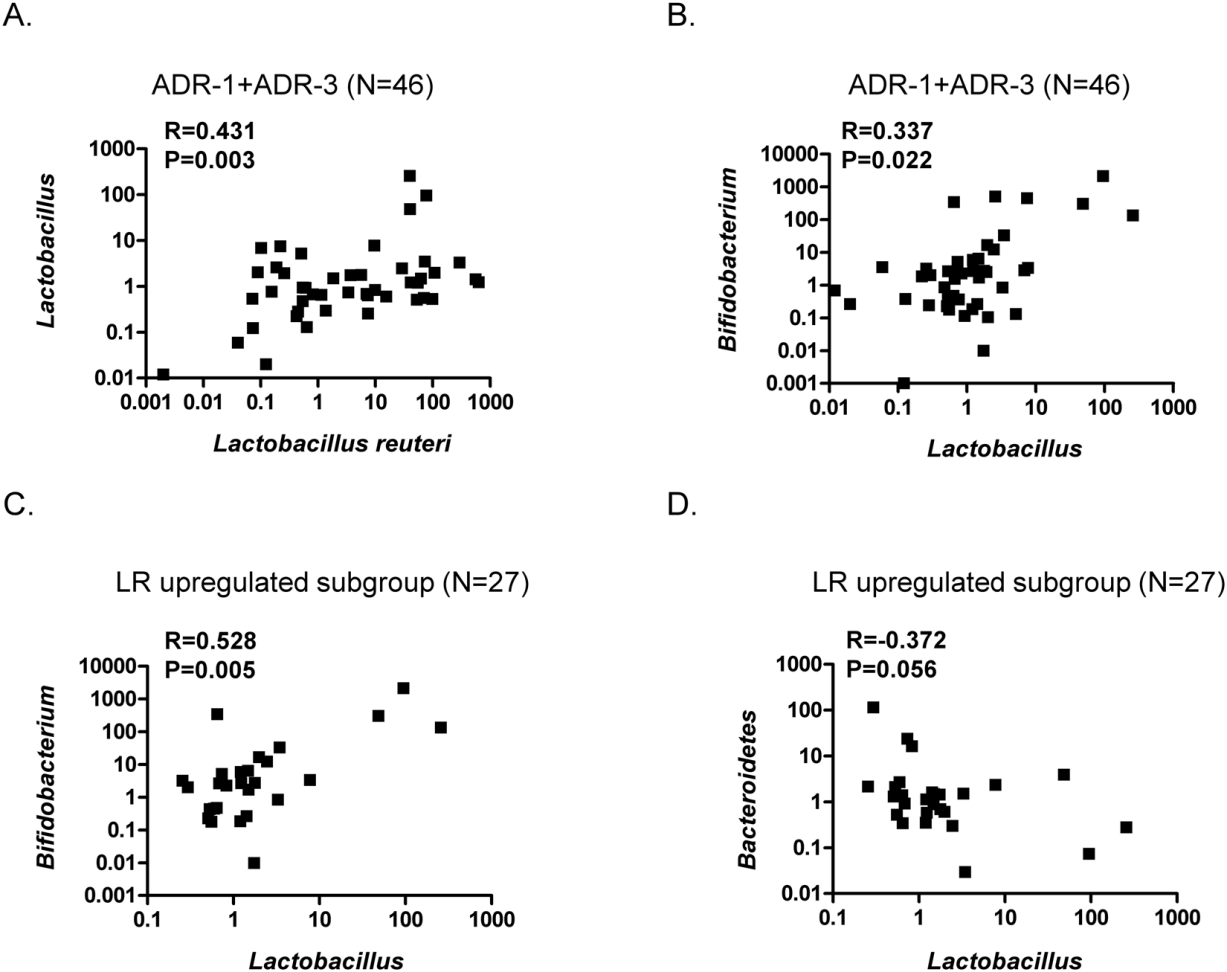
The changes of intestinal flora in probiotics consumption T2DM patients. The association between *L*. *reuteri* and *Lactobacillus spp*. (**A**) or *Lactobacillus spp*. and *Bifidobacterium spp*. (**B**) in probiotics consumption participants (ADR-1 or ADR-3, n = 46). The correlation between *Lactobacillus* and *Bifidobacterium* (**C**) or *Lactobacillus spp*. and *Bacteriodetes* (**D**) in participants with upregulated *L*. *reuteri* (ADR-1 or ADR-3 group, n = 27). The statistical analysis was calculated with Spearman’s rho correlation.

It has been reported that the ratio of *Bacteroidetes* to *Firmicutes* is positively correlated with the serum glucose level of T2DM patients^17^. Metformin, an antidiabetic drug, may increase mucin-degrading *Akkermansia muciniphila*^18,19^. *Bifidobacterium* has been reported to be decreased in diabetic individuals^20^. Consequently, we were interested in using quantitative RT-PCR analysis to investigate the changes in intestinal flora, including *L*. *reuteri*, *Lactobacillus* spp., *Bifidobacterium* spp., *A*. *muciniphila*, *Clostridium* cluster I, *Bacteroidetes*, and *Firmicutes*, after the consumption of ADR-1 or ADR-3 by T2DM participants. We first found that there was an increased level of total *L*. *reuteri* in the ADR-1 and ADR-3 consumption groups in comparison with that in the placebo group, although a significant difference was only observed in the ADR-1 consumption group (Table 3, *p* = 0.017), and the increased total *L*. *reuteri* level in the ADR-1 consumption group was significantly higher than that in the ADR-3 consumption group (Table 3, *p* = 0.022). A significantly increased level of *Bifidobacterium* was observed in the ADR-3 consumption group (Table 3, *p* = 0.049). The levels of *Lactobacillus* spp., *A*. *muciniphila*, *Clostridium* cluster I, *Bacteroidetes*, and *Firmicutes* or the ratio of *Bacteroidetes/Firmicutes* showed no significant differences between the placebo and the ADR-1 or ADR-3 consumption group (Table 3). Correlation analysis was further used to identify the relationship between changes in microbiota and HbA1c changes. The net change in the HbA1c level was significantly negatively correlated with upregulation of total *L*. *reuteri* (Fig. 4A, *p* = 0.025) or *Lactobacillus* spp. (Fig. S3A, *p* = 0.044) and was positively correlated with the *Bacteroidetes/Firmicutes* ratio (Fig. S3B, *p* = 0.009). There was no significant correlation between the net change in the HbA1c level with total *L*. *reuteri* (Fig. 4B, *p* = 0.421), *Lactobacillus* spp. (Fig. S3C, *p* = 0.091), or the *Bacteroidetes/Firmicutes* ratio (Fig. S3D, *p* = 0.181) in participants with downregulation of total *L*. *reuteri*. Furthermore, in the ADR-1 consumption group with upregulated total *L*. *reuteri* (n = 16), the net change in the HbA1c level was significantly positively correlated with *Bacteroidetes* (Fig. S4A, *p* = 0.013) or the *Bacteroidetes/Firmicutes* ratio (Fig. S4B, *p* = 0.001). In the ADR-3 consumption group with upregulated total *L*. *reuteri* (n = 11), the net change in the HbA1c level was significantly negatively correlated with *Lactobacillus* spp. (Fig. S4E, *p* = 0.019). Interestingly, in all participants or in those with upregulated *L*. *reuteri* (n = 27), the reduction in the HbA1c level was observed in only those with a greater than 8-fold increase in upregulated *L*. *reuteri* (Fig. 4C, *p* = 0.034 or Fig. 4D, *p* = 0.033). In participants who consumed probiotics (n = 46), there was a significantly positive correlation between the total *L*. *reuteri* level and *Lactobacillus* spp. (Fig. 5A, *p* = 0.003). The level of *Lactobacillus* spp. was significantly and positively correlated with the *Bifidobacterium* spp. level in participants who consumed probiotics (Fig. 5B, *p* = 0.022). In participants with upregulated *L*. *reuteri* (n = 27), the level of *Lactobacillus* spp. was significantly and positively correlated with *Bifidobacterium* spp. (Fig. 5C, *p* = 0.005), and there was a negative correlation with the *Bacteroidetes* level (Fig. 5D, *p* = 0.056). In participants in the ADR-1 consumption group with upregulated *L*. *reuteri* levels (n = 16), the level of *Lactobacillus* spp. was significantly and positively correlated with *Bifidobacterium* spp. (Fig. S4C, *p* = 0.02) and was significantly and negatively correlated with *Bacteroidetes* (Fig. S4D, *p* = 0.023). In participants in the ADR-3 consumption group with upregulated *L*. *reuteri* levels (n = 11), there was a significant and positive correlation between *Firmicutes* and *L*. *reuteri* (Fig. S4F, *p* = 0.015). In summary, these results suggest that the degree of HbA1c reduction is affected by the level of *L*. *reuteri* upregulation in T2DM patients after ADR-1 or ADR-3 consumption and that there is a difference between ADR-1 and ADR-3 in terms of the change in intestinal flora that may lead to different outcomes for the regulation of blood sugar or T2DM-related complications.

**Table 3:** The changes of intestinal microflora in participates after *L*. *reuteri* consumption.

Microbial groups	Placebo (N = 21)^a,c^	ADR-1 (N = 22)^a^	ADR-3 (N = 24)^a^	*P*-value^b^		
				placebo vs. ADR-1	placebo vs. ADR-3	ADR-1 vs. ADR-3
*Lactobacillus_reuteri*	1.53 ± 1.77	98.43 ± 174.3	6.23 ± 11.25	0.017	0.055	0.022
*Lactobacillus spp*.	2.51 ± 3.66	5.47 ± 20.02	14.53 ± 52.73	0.503	0.277	0.44
*Bifidobacterium spp*.	6.28 ± 19.74	3441 ± 15649	73.72 ± 156.2	0.315	0.049	0.324
*Akkemansia muciniphila*	25.84 ± 59.38	81.07 ± 268.2	170 ± 818.3	0.356	0.398	0.618
*Clostridium* cluster I	20.43 ± 81.26	2.21 ± 2.78	5.57 ± 10.21	0.317	0.415	0.132
*Bacteroidetes*	1.42 ± 1.29	7.21 ± 24.39	2.47 ± 3.53	0.28	0.18	0.38
*Firmicutes*	1.57 ± 2.71	1.62 ± 1.73	3.02 ± 3.35	0.94	0.12	0.08
*Bacteroidetes/Firmicutes*	−99.37 ± 338.03	22.28 ± 166.75	−87.98 ± 213.26	0.15	0.9	0.06

^a^Fecal microflora was analyzed by quantitative PCR as described in “Materials and Methods” section and presented as fold change at 6 month when compared with starting point (mean ± SD).

^b^Two sample *t*-test analysis was used to compare mean values between groups as indicated.

^c^One participate in placebo group lost stool sample.

## Discussion

In this study, we identified another *L*. *reuteri* strain, ADR-1, with beneficial effects on a T2DM rat model, and we conducted a double-blinded, randomized and placebo-controlled clinical trial to investigate the clinically beneficial effects of ADR-1 and ADR-3 on T2DM patients. The results revealed that oral consumption of ADR-1 or ADR-3 by T2DM patients was safe, without any adverse effects. Although there were two cases of severe adverse effects, these were not caused by the consumption of ADR-1 or ADR-3. Our data indicated that the consumption of ADR-1 displayed a reduction effect on serum HbA1c levels, and the intake of ADR-3 showed a beneficial effect on blood pressure reduction (Table 1). Based on previous studies, a reduction in the HbA1c level is often observed in probiotic consumption trials using a blend of probiotic genera, including *Lactobacilli* and *Bifidobacteria*^21–23^, but rare in trials using a single probiotic strain^21,24^. The results for ADR-1 suggest that it has great potential in T2DM management.

Another important finding of our study was the observation that the reduction in HbA1c levels was observed in only subjects with a greater than 8-fold increase in fecal *L*. *reuteri* (Fig. 4D). The difference in outcomes between ADR-1 and ADR-3 regarding the HbA1c level may have resulted from the preparation of these two *L*. *reuteri* strains: ADR-1 was supplied as a live organism but ADR-3 was supplied as a heat-killed organism. From our results, the greater increase in total fecal *L*. *reuteri*, which has been observed to be associated with glycemic control in germ-free mice^25^, was found in participants who consumed live ADR-1 (Table 3). Based on the RAPD and API-50 CHO tests (Fig. S1), it is clear that ADR-1 differs from ADR-3, although the BSH activity of these two strains was similar (Fig. S5). From these results, one suggestion is to increase the dosage of heat-killed ADR-3 that is consumed for glycemic control in T2DM patients. Additionally, a greater than 8-fold increase in the total fecal *L*. *reuteri* level may be used as an indicator for predicting the possible beneficial effects of probiotic products on T2DM management. Although the consumption of live probiotics products is generally considered safe for most populations, some side effects have been reported under certain conditions. For example, live probiotics may become pathogenic when used in subjects with severe immunodeficiency^14^. In infants with short bowel or cardiac stenosis, bacteremia has been reported in some cases^26^. Another advantage of heat-killed probiotics is that they are relatively easy to standardize. To develop heat-killed probiotics products is thus still worthwhile for the health care market.

**Figure 6:**
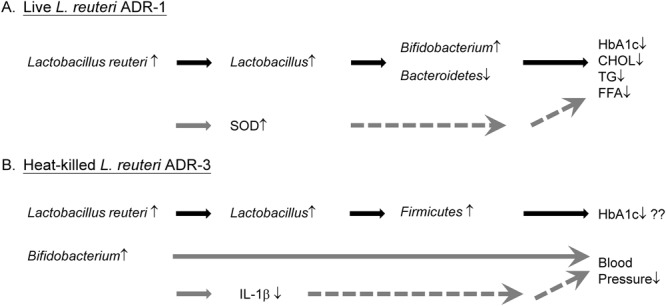
Hypothetical mechanisms of beneficial effect of ADR-1 or ADR-3 consumption in T2DM patients. (**A**) Intake of live ADR-1 could increase intestinal level of *L*. *reuteri* to further up-regulate *Lactobacillus spp*., and *Bifidobacterium spp*. and decrease *Bacteroidetes*. The changes of intestinal microflora may lead to the reduction of HbAlc and blood lipids. The reduction of blood lipids may also result from the up-regulation of serum SOD level. (**B**) Consumption of heat-killed could increase intestinal level of *L*. *reuteri* to further up-regulate *Lactcobacillus spp*. and *Firmicutes* which may let patients to control their HbAlc level. The increased level of *Bifidobacterium spp*. in ADR-3 intake participants may lead to the down-regulation of IL-1β to further reduce blood pressure.

The possible mechanisms of the beneficial effects of *L*. *reuteri* consumption on T2DM management might include (1) a reduction in the levels of pro-inflammatory cytokines, (2) the high bile salt hydrolase (BSH) activity of ADR-1 or ADR-3, and (3) the changes in intestinal microflora composition. It has been proposed that the intake of probiotics might improve the integrity of intestinal epithelium and diminish the Toll-like receptor 4 pathway to reduce pro-inflammatory signaling and to enhance insulin sensitivity^27,28^. In our study, the trend of IL-1β changes tended to be reduced after ADR-1 or ADR-3 consumption, although a significant difference was observed in only the ADR-3 consumption group at V3 (Table 2). Microbial bile salt hydrolase (BSH) has been demonstrated to increase the level of unconjugated bile acids and activate TGR5 to improve insulin sensitivity in animal studies^29^. We also found that ADR-1 or ADR-3 contained high BSH activity in comparison to the two reference strains, *L*. *rhamnosus* and *L*. *plantarum* (Fig. S5). On the other hand, microbial BSH has also been demonstrated to play a role in lowering cholesterol^30^. The high BSH activity of ADR-1 or ADR-3 may also be one of the mechanisms by which cholesterol levels are reduced in participants at the 3-month time point after consumption. However, the blood lipids profile is influenced by many factors, including diet, exercise, and stress^31^. To better understand the effect of ADR-1 on blood lipid management in T2DM patients requires another trial that is controlled for lifestyle factors. It has been demonstrated that administration of *Bifidobacterium* reduces serum lipid levels in high-fat-diet-induced obese rats^32^. In a human study, consumption of yogurt consisting of *Bifidobacterium longum* strain BL1 could reduce serum total cholesterol in subjects with moderate hypercholesterolemia^33^. Here, we hypothesize that the beneficial effects on the reduction in serum levels of HbA1c and lipids in T2DM patients in the ADR-1 consumption group could also be a result of increased levels of *L*. *reuteri* followed by the upregulation of *Lactobacillus* spp. and *Bifidobacterium* spp. but reduction in *Bacteroidetes* (Fig. 6A).

Although the changes in HbA1c levels were not significant in the ADR-3 consumption group, decreased blood pressure, such as SBP and MBP, was observed. In intestinal flora analysis, only *Bifidobacterium* spp. was significantly increased in the ADR-3 consumption group. Indeed, we observed a significantly negative correlation between MBP and the *Bifidobacterium* level (Fig. S6) in all participants. Several reports have demonstrated that the consumption of probiotics may improve blood pressure, especially when the duration of consumption lasts for more than 8 weeks or when the daily consumption dose is more than 10^11^ CFU^34^. On the other hand, IL-1β has been known to be elevated in individuals with hypertension, which leads to renal and vascular inflammation^35^. It also has been demonstrated that metabolites from *Bifidobacterium infantis* displayed anti-inflammatory activity to reduce IL-6 or IL-8 production in IL-1β-stimulated human enterocytes^36^. Additionally, it has been reported that fermented milk or a mixture of probiotics called VSL#3 displayed an anti-hypertension effect. In one study, a fermented milk with three different *Lactobacillus* strains displayed an *in vitro* inhibitory effect on angiotensin-converting enzyme (ACE inhibitor), a key enzyme in blood pressure control^37^. Furthermore, treatment with VSL#3, which contains *Streptococcus thermophilus*, *B*. *longum*, *B*. *breve*, *B*. *infantis*, *L*. *acidophilus*, *L*. *plantarum*, *L*. *casei*, and *L*. *bulgaricus*, prevented endothelial dysfunction in a rat model of hypertension through the improvement of vascular oxidative stress and reduction in the levels of TNF-α, IL-1α and MCP-1 in plasma^38^. Our data regarding the beneficial effect of ADR-3 on reductions in SBP and MBP is the first report to use a single *Lactobacillus* strain to potentially achieve blood pressure control. Our results also suggest that the downregulation of IL-1β through the increased intestinal *Bifidobacterium* spp. level after ADR-3 consumption may lead to the control of blood pressure in T2DM patients (Fig. 6B).

In conclusion, the results from our double-blinded, randomized, placebo-controlled trial demonstrate that the *L*. *reuteri* strains ADR-1 and ADR-3 have beneficial effects on T2DM patients. The changes in intestinal flora after the consumption of different *L*. *reuteri* strains may influence the outcomes. The fecal *L*. *reuteri* level after ADR-1 or ADR-3 consumption may thus be used as a predictor of reduced HbA1c levels in T2DM patients in the future.

## Electronic supplementary material

Supporting information

Trial Protocol

Consort checklist of clinical trial
