# Author Correction: The beneficial effects of *Lactobacillus reuteri* ADR-1 or ADR-3 consumption on type 2 diabetes mellitus: a randomized, double-blinded, placebo-controlled trial

**DOI:** 10.1038/s41598-020-60957-9

**Published:** 2020-02-26

**Authors:** Ming-Chia Hsieh, Wan-Hua Tsai, Yu-Pang Jheng, Shih-Li Su, Shu-Yi Wang, Chi-Chen Lin, Yi-Hsing Chen, Wen-Wei Chang

**Affiliations:** 1 Graduate Institute of Integrative Medicine, China Medical University, Taichung, 40402 Taiwan; 2 Division of Endocrinology and Metabolism, Department of Internal Medicine, Changhua Christian Hospital, Changhua, 500 Taiwan; 3 Research and Development Department, GenMont Biotech Incorporation, Tainan, 74144 Taiwan; 4 Department of Life Sciences, Institute of Biomedical Science, National Chung Hsing University, Taichung, 40249 Taiwan; 5 Department of Biomedical Sciences, Chung Shan Medical University, Taichung, 40201 Taiwan; 6 Department of Medical Research, Chung Shan Medical University Hospital, Taichung, 40201 Taiwan

Correction to: *Scientific Reports* 10.1038/s41598-018-35014-1, published online 14 November 2018

This Article contains errors in the Materials and Methods and the Results section.

In the Materials and Methods section under subheading ‘The recruitment of participants’,

“The exclusion criteria were as follows: pregnancy; the presence of other diseases, including cancers (with the exception of well-controlled benign tumors), kidney failure/dialysis, heart diseases, stroke, or autoimmune diseases; the use of medications, including anti-diabetes drugs, and antibiotics, or other probiotic products <4 weeks before randomization; an AST/ALT level >3-fold the normal range; participation in other clinical trials; and the presence of other medical conditions that might jeopardize compliance with the protocol (e.g., malabsorption syndrome or an inability to take orally administered drugs).”

should read:

“The exclusion criteria were as follows: pregnancy; the presence of other diseases, including cancers (with the exception of well-controlled benign tumors), kidney failure/dialysis, heart diseases, stroke, or autoimmune diseases; the use of medications, including anti-diabetes herbs, and antibiotics, or dietary supplements for diabetes care as well as other probiotic products <4 weeks before randomization; an AST/ALT level >3-fold the normal range; participation in other clinical trials; and the presence of other medical conditions that might jeopardize compliance with the protocol (e.g., malabsorption syndrome or an inability to take orally administered drugs).”

In the Results section under subheading ‘L. *reuteri* strain ADR-1 displayed beneficial effects in a T2DM rat model’.

“Reductions in serum antioxidant proteins such as glutathione reductase (GPX) and superoxide dismutase (SOD) were also observed in HFD rats with oral administration of ADR-1 (Fig. 1D).”

should read:

Recovery of down-regulated expression in glutathione reductase (GPX) and superoxide dismutase (SOD) was observed in HFD rats with oral administration of ADR-1 (Fig. 1D).

