# Supplementary material for: The beneficial effects of Lactobacillus reuteri ADR-1 or ADR-3 consumption on type 2 diabetes mellitus: a randomized, double-blinded, placebo-controlled trial
Source: Sci Rep. 2018 Nov 14;8:16791. doi: 10.1038/s41598-018-35014-1 (PMC6235926; doi:10.1038/s41598-018-35014-1)
Supplement: Supplementary file 1 — Supporting information [file 41598_2018_35014_MOESM1_ESM.pdf]

The beneficial effects of Lactobacillus reuteri ADR-1 or ADR-3 consumption on type 2 diabetes mellitus: a randomized, double-blinded, placebo-controlled trial

Ming-Chia Hsieh, Wan-Hua Tsai, Yu-Pang Jheng, Shih-Li Su, Shu-Yi Wang, Chi-Chen Lin, Yi-Hsing Chen, Wen-Wei Chang

Supporting Information

Figures

A.

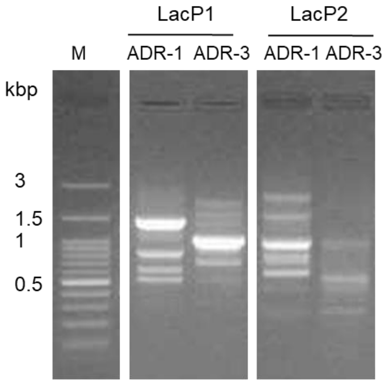

B.

strips no.	carbohydrates substrate	ADR-1	ADR-3	strips no.	carbohydrates substrate	ADR-1	ADR-3	strips no.	carbohydrates substrate	ADR-1	ADR-3
1	Glycerol	-	-	18	D-Mannitol	-	-	35	D-Raffinose	+	+
2	Erythritol	-	-	19	D-Sorbitol	-	-	36	Amidon (starch)	-	-
3	D-Arabinose	-	-	20	Methyl-α-D-mannopyranoside	-	-	37	Glycogen	-	-
4	L-Arabinose	+	+	21	Methyl-α-D-glucopyranoside	-	-	38	Xylitol	-	-
5	D-Ribose	+	-	22	N-Acetyl glucosamine	-	-	39	Gentiobiose	-	-
6	D-Xylose	-	-	23	Amygdalin	-	-	40	D-Turanose	-	-
7	L-Xylose	-	-	24	Arbutin	-	-	41	D-Lyxose	-	-
8	D-Adonitol	-	-	25	Esculin ferric citrate	+	-	42	D-Tagatose	-	-
9	Methyl-β-D-Xylopyranoside	-	-	26	Salicin	-	-	43	D-Fucose	-	-
10	D-Galactose	+	+	27	D-Cellobiose	-	-	44	L-Fucose	-	-
11	D-Glucose	+	+	28	D-Maltose	+	+	45	D-Arabitol	-	-
12	D-Fructose	-	-	29	D-Lactose (bovine origin)	+	-	46	L-Arabitol	-	-
13	D-Mannose	-	-	30	D-Melibiose	+	+	47	Potassium gluconate	+	+
14	L-Sorbose	-	-	31	D-Saccharose (sucrose)	+	+	48	Potassium 2-ketogluconate	-	-
15	L-Rhamnose	-	-	32	D-Trehalose	-	-	49	Potassium 5-ketogluconate	-	-
16	Dulcitol	-	-	33	Inulin	-	-				
17	Inositol	-	-	34	D-Melezitose	-	-				

Fig. S1 ADR-1 and ADR-3 were different strains of *L. reuteri*. (A) RAPD profiles of *L. reuteri* ADR-1 and ADR-3 DNA were amplified by primers of LacP1 (5'- ACGCGCCCT -3' or LacP2 (5'-ATGTAACGCC- 3') using PCR reaction followed by DNA gel analysis. The results showed ADR-1 and ADR-3 were differential strains with individual RAPD profile. (B) Fermentation of carbohydrates ability was determined with API 50 CHL kit. Carbohydrate utilization patterns of ADR-1 or ADP-3 was summarized in this table. These data analyzed by website (https://apiweb.biomerieux.com/jsp/ident/index.jsp) and it was indicated that ADR-1 and ADR-3 were identified as *L. reuteri*. However, ADR-1 fermented CHO ability was different from ADR-3 at strips no.-5、-25、-29, suggesting that they were different strains of *L. reuteri*.

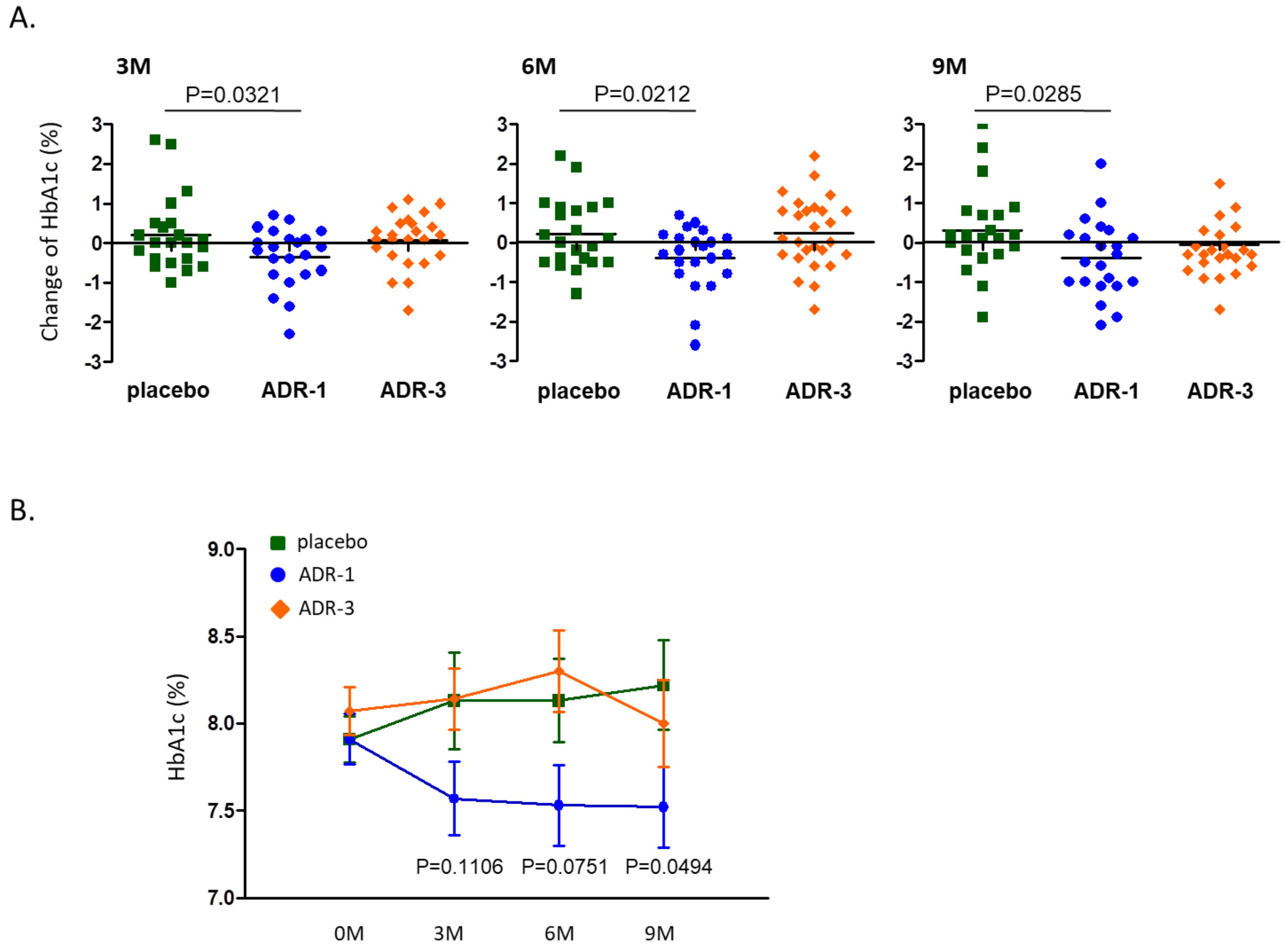

Fig. S2. The serum level of HbA1c among *L. reuteri* consumption or placebo groups. The blood samples of participates were collected at different time point (3, 6 or 9 month) as indicated in Fig. 2B and measured serum HbAlc level. (B) The changes of serum HbA1c level with time in different groups.

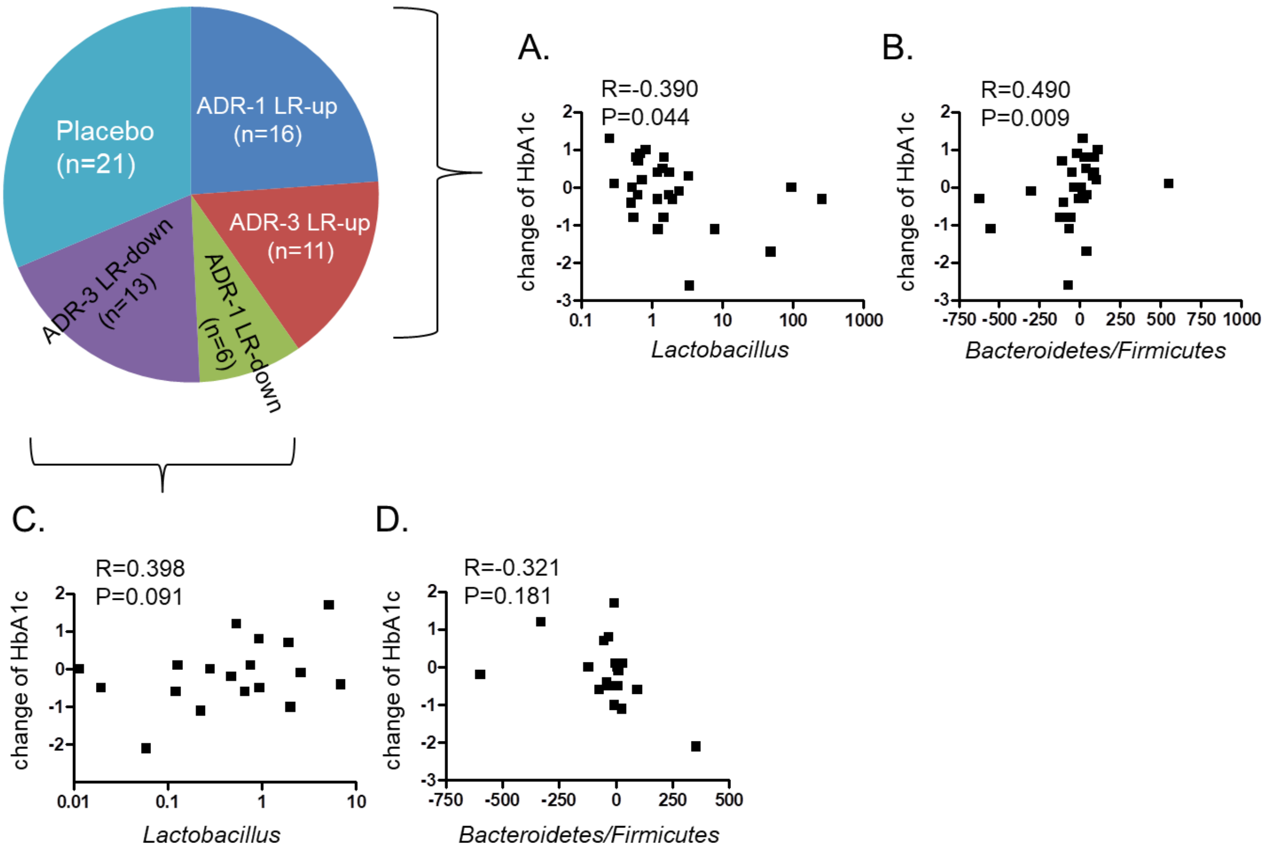

Fig. S3. The association between serum HbA1c level and *Lactobacillus spp.* or ratio of *Bacteroidetes/Firmicutes* among groups with different changes of *L. reuteri* level. The blood and fecal samples of participates at starting time point and 6 month after *L. reuteri* consumption were collected and measured the serum HbAlc level and fecal microflora as described in "Materials and Methods" section. The association between of serum HbAlc level and *Lactobacillus spp.* (A, C), ratio of *Bacteroidetes/Firmicutes* (B, D) in ADR-1 or ADR-3 consumption participates with up-regulated *L. reuteri* level (LR-up, A and B) or with down-regulated *L. reuteri* level (LR-down, C and D) was calculated by Spearman's rho correlation analysis.

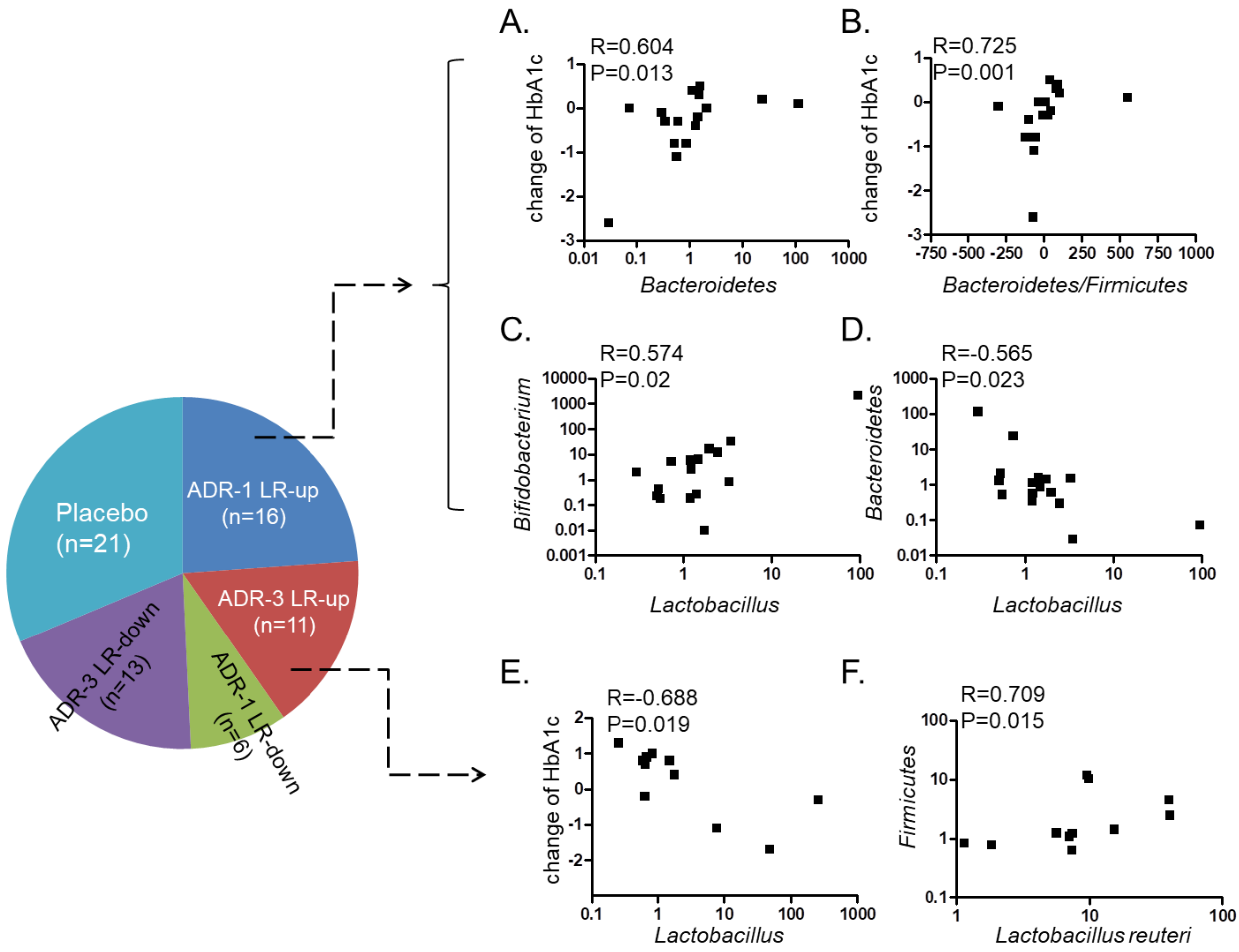

Fig. S4. The association between serum HbAlc level and fecal microbials in ADR-1 or ADR-3 consumption participates with up-regulated *L. reuteri* level. The blood and fecal samples of participates at starting time point and 6 month after *L. reuteri* consumption were collected and measured the serum HbAlc level and fecal microflora as described in "Materials and Methods" section. (A, B) The association between of the chage of serum HbAlc level and *Bacteroidetes* (A) or ratio of *Bacteroidetes/Firmicutes* in ADR-1 intake participates with up-regulated *L. reuteri* level (LR-up). (C, D) The association between Bifidobacterium spp. and Lactobacillus spp. (C) or between *Bacteroidetes* and *Lactobacillus spp.* in ADR-1 intake participates with LR-up was calculated by Spearman's rho correlation analysis. (E, F) The association between the change of serum HbA1c level and Lactobacillus spp. (E) or between *Firmicutes* and *L. reuteri* (F) in ADR-3 intake participates with down-regulated *L. reuteri* level (LR-down) was calculated by Spearman's rho correlation analysis.

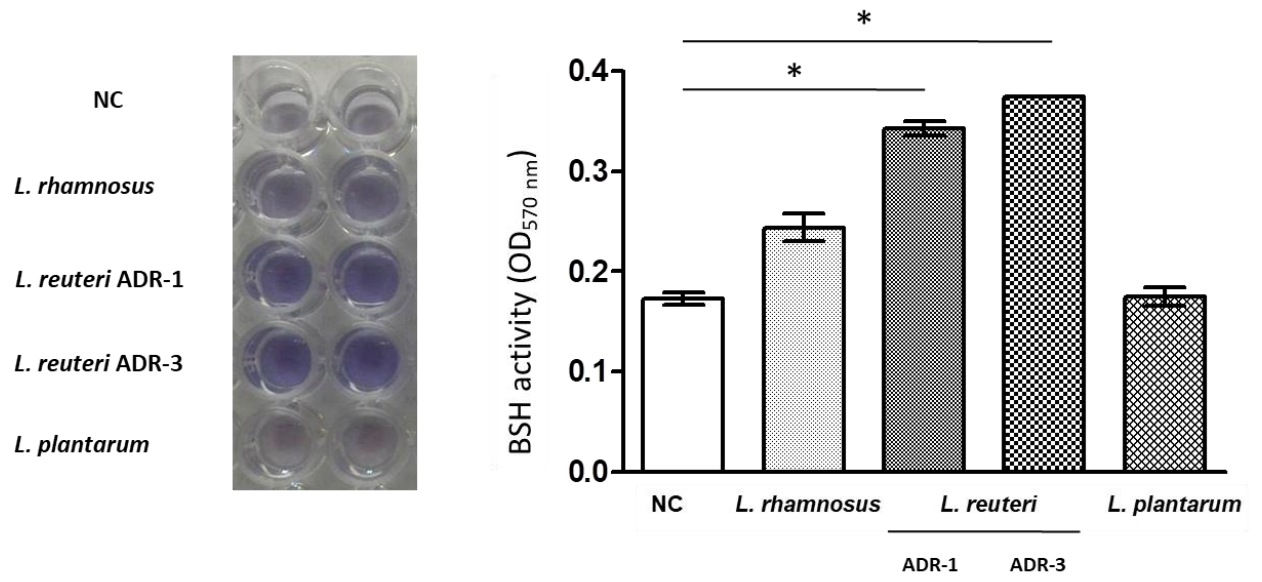

Fig. S5. Bile salt hydrolase (BSH) activity of ADR-1 and ADR-3. Live bacterial culture from *L. rhamnosus*, *L. plantarum*, *L. reuteri* ADR-1 or ADR-3 was used to compare their abilities to de-conjugate bile acids TDCA (Taurodeoxycholic acid sodium salt). After incubation, conjugated bile acids were precipitated with 15% TCA (Trichloroacetic acid) and removed. The unconjugated bile acid was reacted with ninhydrin reagent (left panel) and measured the absorbance at 570 nm wavelength (right panel). The absorbance values indicated the BSH activity of bacteria. NC, negative control (MRS broth). *, $P < 0.05$.

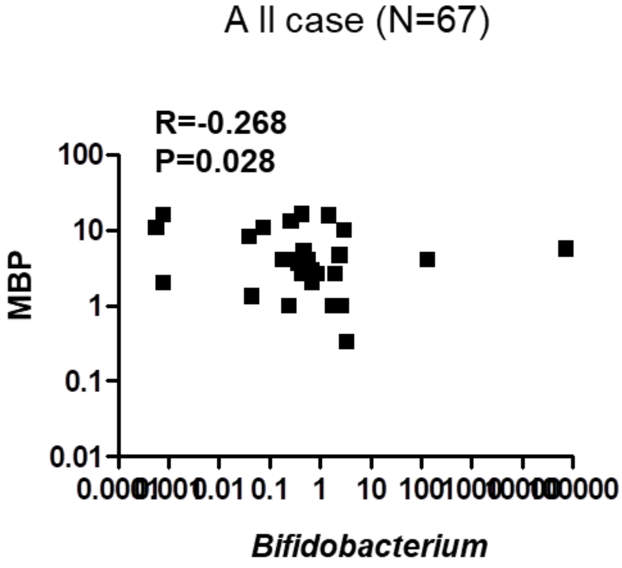

Fig. S6. The association between mean blood pressure (MBP) and fecal level of *Bifidobacterium spp.* in all participates. The fecal samples of all participates (n=67) at starting time point and 6 month after *L. reuteri* or placebo consumption were collected and measured the changes of *Bifidobacterium spp*. level with quantitative PCR method as described in "Materials and Methods" section. The changes of MBP in participates were calculated by the data at 6 month after trial divided by the data at starting point.

Tables

Table S1. Sequences of primer sets used in this study for the determination of fecal microflora.

Target	Name	Sequence (5’-3’)
Lactobacillus spp.	LactoF	TGGAAACAGRTGCTAATACCG
	LactoR	GTCCATTGTGGAAGATTCCC
Bifidobacterium spp.	F-bifido	CGCGTCYGGTGTGAAAG
	R-bifido	CCCCACATCCAGCATCCA
Total bacteria	Total-F	GTGSTGCAYGGYTGTCGTCA
	Total-R	ACGTCRTCCMCACCTTCCTC
Akkermansia muciniphila	AM1-F	CAGCACGTGAAGGTGGGGAC
	AM1-R	CCTTGCGGTTGGCTTCAGAT
Clostridium cluster I	C_clu-F	TACCHRAGGAGGAAGCCAC
	C_clu-R	GTTCTTCCTAATCTCTACGCAT
Lactobacillus reuteri	Hsp_Reuteri_F	GCGTTGATGTTGTTGAAGGAATGAGCTTTG
	Hsp_Reuteri_R	CATCAGCAATGATTAAGAGAGCACGGCC
Bacteroidetes	Bfr230F	CTGAACCAGCCAAGTAGCG
	Bfr230R	CCGCAAACTTTCACAACTGACTTA
Firmicutes	Firm200CF	TGAAACTCAAAGGAATTGACG
	Firm200CR	ACCATGCACCACCTGTC

Table S2. Characteristics of study participates at baseline[a].

	Placebo	ADR-1		ADR-3	
Baseline	N=22	N=22	P-value [b]	N=24	P-value [b]
Male	13 (59.1%)	12 (54.5%)	0.7609	13 (54.2%)	0.7365
Female	9 (40.9%)	10 (45.5%)		11 (45.8%)	
Age	55.77±8.55	52.32±10.20	0.2302	53.88±7.78	0.4346
Height (cm)	161.80±7.28	163.00±7.92	0.6173	162.00±7.83	0.9176
Weight (kg)	72.40±11.64	74.97±15.73	0.5408	73.77±12.54	0.7043
BMI (kg/m^2)	27.53±3.15	28.04±4.29	0.6587	28.03±3.88	0.6374
SBP (mmHg)	126.80±9.93	126.20±13.08	0.8668	132.90±16.93	0.1378
DBP (mmHg)	75.32±9.09	76.68±8.87	0.6172	76.08±6.93	0.7484
Waist circumference (cm)	95.45±10.59	95.94±12.00	0.8873	96.19±9.00	0.7998
Hip circumference (cm)	100.00±7.66	101.90±10.86	0.5073	101.10±7.95	0.6476
AST (U/L)	31.73±14.65	30.91±10.39	0.8319	32.38±17.84	0.8941
ALT (U/L)	39.59±23.11	38.77±15.88	0.8918	43.71±27.67	0.5885
HbA1c (%)	7.91±0.62	7.91±0.68	>0.999	8.07±0.67	0.4252

Abbreviations: BMI, body mass index; SBP, systolic blood pressure; DBP, diastolic blood pressure; AST, aspartate aminotransferase; ALT, alanine aminotransferase.

[a] All the data were collected at first visit.

[b]Chi-quare test (for gender) or two sample *t*-test analysis (for other factors) was used to compare mean values between placebo and ADR-1 or ADR-3 groups.

Table S3. Medical history of study participates[a].

Variable	placebo (N=22)	ADR-1 (N=22)	P-value[b]	ADR-3 (N=24)	P-value[b]
Medical History					
Hypercholesterolemia	18 (81.8%)	18 (81.8%)	>0.99	22 (91.7%)	0.41
Hypertension	16 (72.7%)	18 (81.8%)	0.72	17 (70.8%)	>0.99
Medication for diabetes					
Insulin	5 (22.7%)	3 (13.6%)	0.70	5 (20.8%)	>0.99
Metformin	20 (90.9%)	20 (90.9%)	>0.99	22 (91.7%)	>0.99
Sulphonylurea	16 (72.7%)	14 (63.6%)	0.75	18 (75.0%)	>0.99
DDP-4 inhibitor					
Sitagliptin	4 (18.2%)	2 (9.1%)	0.66	2 (8.3%)	0.41
Vildagliptin	2 (9.1%)	3 (13.6%)	>0.99	2 (8.3%)	>0.99
Saxagliptin	12 (54.6%)	9 (40.9%)	0.55	10 (41.7%)	0.56
Linagliptin	7 (31.8%)	5 (22.7%)	0.74	8 (33.3%)	>0.99
GLP-1 Receptor agonists					
Exenatide	0 (0.0%)	1 (4.6%)	>0.99	2 (8.3%)	0.49
Liraglutide	0 (0.0%)	3 (13.6%)	0.23	2 (8.3%)	0.49
Acarbose	6 (27.3%)	5 (22.7%)	>0.99	2 (8.3%)	0.13
Medication for Hypertension					
Diuretics	6 (27.3%)	4 (18.2%)	0.72	8 (33.3%)	0.75
Beta-blockers	6 (27.3%)	7 (31.8%)	>0.99	9 (37.5%)	0.54
Alpha/ Beta-blockers	0 (0.0%)	1 (4.6%)	>0.99	0 (0.0%)	NA
ACEI	0 (0.0%)	0 (0.0%)	NA	1 (4.2%)	>0.99
AII RA	13 (59.1%)	8 (36.4%)	0.23	9 (37.5%)	0.24
CCB	1 (4.6%)	1 (4.6%)	>0.99	1 (4.2%)	>0.99
Vasodilator	2 (9.1%)	0 (0.0%)	0.49	3 (12.5%)	>0.99
Mixed-Drug	6 (27.3%)	11 (50.0%)	0.22	9 (37.5%)	0.54

Abbreviations: ACEI, Angiotensin-Converting Enzyme Inhibitor; AII RA, Angiotensin II Receptor Antagonist; CCB, Calcium channel blockers.

[a] Data were collected with medical records at first visit.

[b] Fisher exact test was used to compare mean values between placebo and ADR-1 or ADR-3 groups.
