# Supplementary material for: The beneficial effects of Lactobacillus reuteri ADR-1 or ADR-3 consumption on type 2 diabetes mellitus: a randomized, double-blinded, placebo-controlled trial
Source: Sci Rep. 2018 Nov 14;8:16791. doi: 10.1038/s41598-018-35014-1 (PMC6235926; doi:10.1038/s41598-018-35014-1)
Supplement: Supplementary file 2 — Trial Protocol [file 41598_2018_35014_MOESM2_ESM.pdf]

Study Protocol

PROTOCOL NO.: P201404-31
STUDY DRUG: ADR-1 and ADR-3 capsules
ClinicalTrials.gov Identifier：NCT02274272
INDICATION: Type 2 Diabetes
FIRST RECORDED DATE: **Oct-24-2014**

**TITLE**	Effects of Genmont Probiotic on Improve the Level of Blood Glucose and Other Diabetic Associate Parameter in Type 2 Diabetes Patients.
**PRINCIPAL INVESTIGATOR**	Ming-Chia Hsieh, M.D.<br>Assistant Administrator, Diabetes E Institute<br>Changhua Christian Hospital, Changhua, Taiwan<br>Yu-Pang Jheng, M.D.<br>Attending Physician, Division of Metabolism & Endocrinology<br>Changhua Christian Hospital, Changhua, Taiwan<br>Shu-Yi Wang, M.D.<br>Attending Physician, Division of Metabolism & Endocrinology<br>Changhua Christian Hospital, Changhua, Taiwan<br>Shih-Li Su, M.D.<br>Chief Physician, Division of Metabolism & Endocrinology<br>Changhua Christian Hospital, Changhua, Taiwan
**SPONSOR**	GenMont Biotech Inc.
**VERSION/DATE**	**Version 7.0 Sep-03-2014 (Last version v11.0 Sep-02-2016)**

CONFIDENTIAL

The information in this document is confidential and shall remain the exclusive property of **GenMont Biotech Inc.**. The use of such confidential information will not be disclosed to parties not associated with the clinical investigation or used for any purpose without written authorization from **GenMont Biotch Inc.**.

TABLE OF CONTENTS

1. INTRODUCTION..8

2. STUDY OBJECTIVES AND ENDPOINTS..9

2.1 Study Objectives..9

2.2 Endpoints..10

3. STUDY DESIGN..122

4. SELECTION OF SUBJECTS..144

4.1 Number of Subjects..145

4.2 Inclusion Criteria..144

4.3 Exclusion Criteria...144

4.4 Withdrawal Criteria..155

5. TREATMENT PLAN..155

5.1 Description of Study Agent..155

5.2 Study Drug...166

5.3 Study Assessment..18

5.4 Duration of Treatment..21

5.5 Concomitant Medication or Treatment.......................................211

6. STUDY MEASUREMENT AND VARIABLES..............................211

6.1 Patient Characteristics...211

6.2 Study Procedures...222

6.3 Clinical Efficacy Assessments..27

6.4 Clinical Safety Assessments...28

7. DISCONTINUATION OF SUBJECTS..28

8. ADERSE EVENTS (AE) AND SERIOUS ADERSE EVENTS(SAE)
…………………………………………………………………………….28

8.1 Definition of AE...錯誤! 尚未定義書籤。

8.2 Definition of SAE...29

8.3 Clinical Laboratory Abnormalities ....錯誤! 尚未定義書籤。

8.4 Reporting and Recording of AE and SAE.....錯誤! 尚未定義書籤。

9. DATA MANAGEMENT AND EVALUATION 32
9.1 Data Management 32
9.2 Sample Size 33
9.3 Analyzed Population 33
9.4 Statistical and Analytical Methods 33
9.5 Premature Termination and Missing Values 36
10. ETHICAL AND LEGAL ASPECTS 36
10.1 Local Regulations / Declaration of Helsinki 36
10.2 Independent Ethics Committees / Institutional Review Board 36
10.3 Subject Information and Informed Consent 37
10.4 Subject Data Protection 37
10.5 Protocol Amendments 38
11. STUDY DOCUMENTATION 38
11.1 Source Documentation and Records Retention 38
11.2 Source Documents and Background Data 38
11.3 Case Report Form (CRF) 39
12. REFERENCES 40

SYNOPSIS

**Sponsor:** GenMont Biotech Inc.	**Protocol No.:** P201404-31
**Brand Name:** ADR-1 and ADR-3 capsules	**Active Ingredient:** *Lactobacillus reuteri* ADR-1 and *Lactobacillus reuteri* ADR-3

**Title**	Effects of Genmont Probiotic on Improve the Level of Blood Glucose and Other Diabetic Associate Parameter in Type 2 Diabetes Patients.
**Principal Investigator**	Ming-Chia Hsieh, M.D.<br>Yu-Pang Jheng, M.D.<br>Shu-Yi Wang, M.D.<br>Shih-Li Su, M.D.
**Study Center**	Changhua Christian Hospital, Changhua, Taiwan
**Clinical Phase**	Phase II
**Objective**	1. To evaluate the efficacy profile of probiotics *Lactobacillus reuteri* GMNL-89 (ADR-1) and *Lactobacillus reuteri* GMNL-263 (ADR-3) in patients with Type 2 Diabetes<br>2. To evaluate the safety profiles of probiotics *Lactobacillus reuteri* GMNL-89 ADR-1 (ADR-1) and *Lactobacillus reuteri* GMNL-263 (ADR-3) in patients with Type 2 Diabetes
**Sample Size**	Approximately 120 subjects will be enrolled for treatment in order to collect at least 90 evaluable subjects.
**Inclusion and Exclusion Criteria**	**Inclusion Criteria**<br>1. Subjects in age of 25-70 years old<br>2. Subjects with a history of type 2 diabetes for at least 6 month.<br>3. Subject's glycated hemoglobin (HbA1c) throughout the screening period should be 7 % < HbA1c ≦ 10 %; at least 6 days should be recorded during the screening period.

**Sponsor:** GenMont Biotech Inc.	**Protocol No.:** P201404-31
**Brand Name:** ADR-1 and ADR-3 capsules	**Active Ingredient:** *Lactobacillus reuteri* ADR-1 and *Lactobacillus reuteri* ADR-3

	4. Subject's body mass index (BMI) should be ＞18.5.<br>**Exclusion Criteria**<br>1. Subjects are pregnant, lactating or planning to become pregnant.<br>2. Subjects with any other serious diseases such as cancer (patient with benign tumor under medical control should not be ruled out), kidney failure / dialysis, heart disease, stroke.<br>3. Subjects with Autoimmune Disease.<br>4. Administration of other healthy food for diabetes 4 weeks before intervention.<br>5. Administration of probiotic 4 weeks before intervention.<br>6. Administration of antibiotics 4 weeks before intervention.<br>7. Participation in other clinical trials.<br>8. Subjects with ALT/SGPT or AST/SGOT > 3x upper limit of normal (ULN)<br>9. Subjects with estimated Glomerular filtration rate (eGFR) <30mL/min/1.73m2.<br>10. Subjects who is lack of physical integrity of gastrointestinal tract, or malabsorption syndrome, or inability to take oral medication.
**Study Agent**	ADR-1 and ADR-3 capsules
**Dosage**	Subjects who meet all eligible requirements for entering the study will be followed by randomization into either treatment groups or control group with a ratio of 1:1:1 as shown below:<br>1. Group A： Subjects received Placebo, Two placebo capsules, once daily, QD.<br>2. Group B： Subjects received ADR-1, Two capsules with 4×10^9 colony forming unit (cfu) *Lactobacillus reuteri* GMNL-89, once

	daily, QD.<br>3. Group C： Subjects received ADR-3, Two capsules with 2×10^{10} cells *Lactobacillus reuteri* GMNL-263, once daily, QD.
**Study structure**	Double-blind, parallel, randomized, placebo-controlled
**Duration**	9 months (6 months for intervention and 3 months for follow-up)
**Endpoints**	**Primary Outcome**<br>1. Change in blood sugar profile [Time Frame: 6 months]<br>Fasting blood samples at baseline and after intervention will be collected. Blood sugar profile including sugar (mg/dl) and HbA1c (%) will be assessed.<br>2. Change in blood lipid profile [Time Frame: 6 months]<br>Fasting blood samples at baseline and after intervention will be collected. Blood lipid profile including cholesterol (mg/dl), triglycerides(TG, mg/dl), high density lipoprotein (HDL, mg/dl), low density lipoprotein (LDL, mg/dl) will be assessed.<br>**Secondary Outcome**<br>1. Change in the insulin resistance and inflammatory marker [Time Frame: 6 months]<br>Fasting blood samples at baseline and after intervention will be collected. Insulin resistance and inflammatory marker insulin (μIU/ml), Homeostasis model assessment for insulin resistance (HOMA-IR, ratio) C-peptide (ng/ml) and cytokine will be assessed.<br>2. Change in the gut microbiota [Time Frame: 6 months]<br>Stool samples at baseline and after intervention will be collected. Gut microbiota profile will be assessed.

**Sponsor:** GenMont Biotech Inc.	**Protocol No.:** P201404-31
**Brand Name:** ADR-1 and ADR-3 capsules	**Active Ingredient:** *Lactobacillus reuteri* ADR-1 and *Lactobacillus reuteri* ADR-3

Statistical Methods

Data Analysis

Efficacy Endpoint Analysis

Basic informations were analyzed using the Chi-quare test or two sample t-test. Medical history, medication for diabetes or hypertension in differential groups was analyzed using Fisher exact test. Comparisons of the results change between the probiotic and placebo groups were analyzed using the Two sample t-test. Correlation between the variables was computed by Spearman's rho correlation provided by PASW Statistics 18 Software (SPSS Inc).

Safety Endpoint Analysis

In the analysis of adverse events, the Medical Dictionary for Regulatory Activities (MedDRA) adverse event dictionary will be used to map verbatim adverse events to preferred terms and system organ class. The tabulations will count the number of subjects reporting individual adverse events. In addition, the incidence of adverse events will be categorized by severity and related to the time of occurrence. Changes in physical examinations, changes in laboratory test results (hematology, biochemistry and urinalysis tests) from screening period, and change of vital signs will be summarized and analyzed by the descriptive statistics.

1. INTRODUCTION

Type 2 diabetes mellitus (Type 2 DM) has become one of the fastest growing public health problems in both developed and developing countries. The number of people suffering from diabetes in the world is as high as 135 million to 160 million. Diabetes has a high sugar concentration in blood and urine, however, the most terrible thing about diabetes is that it causes complications such as retinopathy, kidney disease, neurological disorders, and even brain and circulatory disorders. There are basically three ways to treat diabetes: (1) diet therapy (2) exercise therapy (3) drug therapy, among them, dietary therapy is the most basic and most important therapy. Dietary control can help increase the effectiveness of medications for diabetes.

In recent years, more and more researchers have devoted themselves to the study of alternative therapies for diabetes. Among these alternative therapies, the most promising is the use of probiotics to improve diabetes. Present studies have demonstrated that oral administration of probiotics can improve diabetes related symptoms (Yun, S. I. *et. al.* 2009, Yadav, H. *et. al.* 2008, Membrez, M. *et. al.* 2008, Ejtahed HS *et. al.* 2012). The literature indicated that the distribution of flora in the gastrointestinal tract of type 2 diabetes is significantly different from that of normal people (Larsen N *et. al.* 2010).

In patients with diabetes, excessive glucose accumulation can induced reactive oxygen species (ROS) and inflammation cytokines over-produced (Lee, H. B. *et. al.* 2003, Ha, H. and Lee, H. B. 2003). Clinical studies have demonstrated that probiotic yogurt consumption could significantly improve fasting blood glucose, antioxidant and inflammation indicators in Type 2 diabetes patients (Ejtahed HS *et. al.* 2012, Mazloom Z *et. al.* 2013). The clinical study also found that probiotic yogurt can help insulin concentration maintenance in pregnant women(Asemi Z *et. al.* 2013). In prelimary study and published data (Hsieh FC *et. al.* 2013), STZ intraperitoneally treated SD rats model were used to assess the probiotic strains from Genmont biotech Inc. could improve metabolic-related symptoms and complications of diabetes. *L. reuteri* GMNL-89 and GMNL-263 could regulate blood sugar level in STZ treated rat. The study showed that the prognosis of patients with diabetes had highly correlation with blood IL-10 (Kung WJ *et. al.* 2010). We also found that *L. reuteri* GMNL-89 could stimulate IL-10 production in SD rat. Reports showed that probiotic products could improve blood

lipid, including total cholesterol, LDL (Ejtahed HS *et. al.* 2011) and HDL level (Moroti C *et. al.* 2012) in Type II diabetes patients. Our results also found that *L. reuteri* GMNL-89 and GMNL-263 (ADR-3) could regulate blood lipid level in STZ treated rat. The serum levels of GOT, GPT, and γ-GT in diabetic rats was significantly decreased after *L. reuteri* GMNL-89 or GMNL-263 (ADR-3) feeding. Previously study found that consumption *L. reuteri* GMNL-263 (ADR-3) can also improve renal fibrosis in type 1 diabetic rats (Lu YC , *et. al.* 2010).

Based on these results, *L. reuteri* GMNL-89 or GMNL-263 could improve the symptoms and complications in diabetic rat. It also implied these probiotics could reduce the dose of medicines used and avoid the occurrence of side effects. Oral administration of *L. reuteri* GMNL-89 or GMNL-263 (ADR-3) for 28 days in rats also demonstrated its safety. So far, no adverse reactions or serious customer complaints have been received from any consumer. The purpose of this study is to evaluate the efficacy and safety of probiotics ADR-1/GMNL-263 capsules (*Lactobacillus reuteri* ADR-1/*Lactobacillus reuteri* ADR-3) for the treatment of adults with type 2 DM.

2. STUDY OBJECTIVES and ENDPOINT

2.1 Study Objectives

2.1.1 Primary Objective

To evaluate the efficacy of consumption of probiotics *Lactobacillus reuteri* GMNL-89 (ADR-1) or *Lactobacillus reuteri* GMNL-263 (ADR-3) on Type 2 DM patients with medical treatments. The main assessment items include: HbA1c、blood sugar、blood lipid、biomarkers of inflammation and intestinal microbiota profiles.

2.1.2 Secondary Objective

To evaluate the safety assessment of consumption of probiotics *Lactobacillus reuteri* GMNL-89 (ADR-1) and *Lactobacillus reuteri* GMNL-263 (ADR-3) on Type 2 DM patients with medical treatments. The safety features are determined through the changes of vital signs, physical examinations, laboratory test result (hematology, biochemistry and urinalysis tests), as well

as the occurrence of adverse event.

2.2 Endpoints

2.2.1 Primary Endpoint

The change level between baseline and 6th month treatment of blood sugar and blood lipid will be analyzed to assess the effect of daily intake of *L. reuteri* ADR-1 and ADR-3 on type 2 DM subject's treatment.

2.2.2 Secondary Endpoints

Efficacy

The change level between baseline and 6th month treatment of insulin resistance related markers、inflammatory makers and cytokines、antioxidant index and gut microbiota profile will be assessed to determine the effect of daily intake of *L. reuteri* ADR-1 and ADR-3 on type 2 DM subjects treatment.

Safety

The endpoints used to achieve the secondary objectives of the study in safety profiles are listed below:

➢ **Change of vital signs**

The vital signs, including blood pressures, pulse rate, respiratory rate and body temperature, will be assessed for change compared with baseline.

➢ **Changes of physical examinations**

Physical examinations including the following items will be performed at the baseline, 3rd and 6th month visits:

- Skin
- Eyes
- Ear, nose, and throat
- Cardiovascular system

- Respiratory system
- Gastro-intestinal system
- Blood and blood forming organs
- Mental status
- Other system

➢ **Changes of hematology tests**

The hematology tests including the following items will be performed at the baseline, 3rd and 6th month visit:

- White blood cell differential count	- Platelet
- White blood cells	- MCV
- Red Blood cells	- MCH
- Hemoglobin	- MCHC
- Hematocrit	- RDW

➢ **Changes of biochemistry tests**

The biochemistry tests including the following items will be performed at the baseline, 3rd and 6th month visit:

- Aspartate transaminase (SGOT or AST)
- Alanine aminotransferase (SGPT or ALT)
- Blood urea nitrogen (BUN)
- Blood Creatinine

➢ **Changes of urinalysis tests**

The urinalysis tests including the following items will be tested at the the baseline, 3rd and 6th month visit visits:

- White blood cells	- Ketone body
- Red blood cells	- Urine micro albumin
- pH	- Urine creatinine
- Protein	- Glomerular filtration rate (GFR)
- Glucose	- Urine albumin / creatinine ratio (ACR)

➢ **The occurrence of adverse event**

Adverse event will be recorded at each visit starting from Visit 1.

3. STUDY DESIGN

The design of this study bases on the results of studies using animal models. The study is designed for the establishment of the efficacy and safety profile of this investigational product (ADR-1 and ADR-3) for patients with type 2 diabetes based on a double-blind, parallel, placebo-control setting. The duration of the treatment is 6 months, which is recommended by the results of animal testing (*L. reuteri* ADR-1 4×10^9 cfu/day and ADR-3 2×10^{10} cells/day attained its effectiveness at week-12 visit in downregulating the glycated hemoglobin level). The flow chart of the study (as shown below) is also basically based on the design of animal experiments.

Flow chart of the screening, treatment and follow-up period:

SCREENING	TREATMENT			FOLLOW-UP
Visit 0<br>Day -10	Visit 1<br>Baseline	Visit 2<br>3rd Month	Visit 3<br>6th Month	Visit 4<br>9th Month

N=120 → **R** →

- Control group: placebo, N=40
- Treatment group: *L. reuteri* GMNL-89 (ADR-1), N=40
- Treatment group: *L. reuteri* GMNL-263 (ADR-3), N=40

Study Flow Chart

Procedures to be done	Screening visit	Randomization visit	Evaluation visit		Final visit
**Visit**	0	1	2	3	4
**Month**	-	0	3	6	9
**Day**	-10	0	90 (-10~+0)	180 (-10~+7)	270 (±10)
Informed consent form signed/ given	X				
Inclusion and exclusion criteria	X	X			
Weight and Height	X	X	X	X	X
Waistline and Hip circumference	X	X	X	X	X
Demographic data[a]	X				
Medical history (including history of diabetes mellitus)	X				
Urine pregnancy test (applicable only)	X				
Vital signs[b]	X	X	X	X	X
Physical examinations[c]	X	X	X	X	X
Hematology tests[d]	X		X	X	
Biochemistry tests[e]	X		X	X	X[j]
Urinalysis tests[f]	X		X	X	
Inflammatory markers/ Cytokines[g]	X		X	X	
Antioxidant index[h]	X		X	X	
Subject's stool collected		X		X	
Previous medication history[i]	X				
Subject identifier assigned	X				
Subject randomization number assigned		X			
Diet recorded by subject	X				
Dispense diet and exercise record card (DERC)		X	X	X	
Retrieve and review subjects' DERC		X	X	X	X
Record concomitant medication	X	X	X	X	X
Dispense trial medication		X	X	X	
Retrieve unused medication			X	X	X
Record adverse events (AE)		X	X	X	X
Complete exit form					X

a Demographic data includes: date of birth and gender

b Vital sign measurements include: blood pressure, pulse rate, respiratory rate and body temperature

c Physical examinations include: Skin, eyes, ear, nose, throat, cardiovascular system, respiratory system, Gastro-intestinal system, blood and blood forming organs, metal status and other system

d Test items include: a. White blood cell differential count, b. White blood cells, c.Red blood cells, d. Hemoglobin, e.

Hematocrit, f. Platelet, g. MCV, h. MCH, i. MCHC, j. RDW

e Test items include: a. AST, b. ALT, c. Blood urea nitrogen (BUN) d. Blood Creatinine, e. Triglyceride (TG), f. Cholesterol (CHOL), g. Low-density cholesterol (LDL-C), h. High-density lipoprotein cholesterol (HDL-C), i. LDL-C/ HDL-C, j. CHO/HDL, k. Fasting plasma glucose (FPG), l. Glycated hemoglobin (HbA1c), m. Insulin, n. C- peptide, o. Insulin resistance (HOMA-IR), p. Free fatty acid (FFA)

f Test items include: a. White blood cells, b. Red blood cells, c. pH, d. Protein, e. Glucose, f. Ketone body, g. Urine micro albumin, h. Urine creatinine, i. Glomerular filtration rate (GFR), j. Urine albumin / creatinine ratio (ACR)

g Test items include: a. C-reactive protein (CRP), b. Interleukin 6 (IL-6), c. Tumor necrosis factor alpha(TNFα), d. Interleukin-10 (IL-10), e. Interleukin-17 (IL-17), f. Interleukin-1β(IL-1β)

h Test items include: a. Glutathione peroxidase (Gpx), b. Superoxide dismutase (SOD)

i Previous medications of DM should be recorded up to 6 months prior to the first investigational product administration. Other medications should only be recorded up to 1 month before the subjects enter the study.

j Only Glycated hemoglobin (HbA1c) at the 4th visit

4. SELECTION OF SUBJECTS

4.1 Number of Subjects

With a type I error of 5% and a power of 80%, this study requires 90 evaluable subjects. As a dropout rate of 25% is considered, approximately 120 subjects are planned to be enrolled into this study to complete a total of 90 evaluable subjects.

4.2 Inclusion Criteria

1. Subjects in age of 25-70 years old
2. Subjects with a history of type 2 diabetes for at least 6 month
3. Subject’s glycated hemoglobin (HbA1c) throughout the screening period should be 7 % < HbA1c ≦ 10 %; at least 6 days should be recorded during the screening period.
4. Subject’s body mass index (BMI) should be ＞18.5.

4.3 Exclusion Criteria

1. Subjects are pregnant, lactating or planning to become pregnant.
2. Subjects with any other serious diseases such as cancer (patient with benign tumor under medical control should not be ruled out), kidney failure / dialysis, heart disease, stroke.
3. Subjects with Autoimmune Disease.

4. Administration of other healthy food for diabetes 4 weeks before inclusion.
5. Administration of probiotic 4 weeks before inclusion.
6. Administration of antibiotics 4 weeks before inclusion.
7. Participation in other clinical trials.
8. Subjects with ALT/SGPT or AST/SGOT > 3x upper limit of normal (ULN)
9. Subjects with estimated Glomerular filtration rate (eGFR) <30mL/min/1.73m2.
10. Subjects who is lack of physical integrity of gastrointestinal tract, or malabsorption syndrome, or inability to take oral medication.

4.4 Withdrawal Criteria

The subject should be withdrawn from the study if any of the conditions set below occurs:

1. Subject or his/her legally acceptable representative decides to withdraw his/her informed consent form.
2. Investigator considers that the subject is no longer physically and/or psychologically feasible to be included in the study.
3. Subject with poor drug compliance, defined as less than 75% mean drug compliance (See **Section 5.2.7**).
4. Adverse effects occur in subject that the investigator considers a permanent cessation of the study treatment is necessary.

The investigator should complete all the evaluation items at Visit 4 upon a subject's withdrawal. It does not include a subject who has not been treated with the study drug, such as a withdrawal at Visit 0 or 1. Only the CRF required data should be completed and the study drug(s) that has been dispensed to the subject must be retrieved.

5. TREATMENT PLAN

5.1 Description of Study Agent

The investigational product (IP) for this study is supplied by GenMont Biotech Inc.. The sponsor is responsible to ship the IP to each study sites as well as strict inventorial control for the drug accountability implemented.

5.2 Study Drug

5.2.1 Dose and Administration

Each subject will be instructed to take two capsules per day orally with cold or warm water before eating the meal in the morning. No dose modification plan will be anticipated for this study.

5.2.2 Rationale of Dosage

Based on the previous study demonstrated that the reductions of glycosylated hemoglobin (HbA1c), lipid profiles and the improvement of glucose tolerance in *L. reuteri* GMNL-89 (ADR-1) 4×10^9 cfu/d and *L. reuteri* GMNL-263 (ADR-3) 2×10^{10} cells/day at week-12 were significant in the animal modle. Therefore, *L. reuteri* GMNL-89 (ADR-1) 4×10^9 cfu/d and *L. reuteri* GMNL-263 (ADR-3) 2×10^{10} cells/day could be selected as the optimal dosage in the treatment of patients with DM in this study.

5.2.3 Treatment Assignment

Subjects become eligible at Visit 1 will be randomized to treatment groups on a 1:1:1 basis between *L. reuteri* ADR-1, GMNL-263 and placebo. The treatment assignment process will be in a double blinded fashion. Stratified randomization will be employed to prevent the distribution of treatments unbalanced. Subjects will be separated into three groups. For allocation of the participants, a computer-generated list of random numbers was used.

5.2.4 Packaging and Labeling

The IP will be packaged as capsules with appearance identical in all aspects. The capsules will be packaged in bottle or blister and placed in a box for each dispensation at Visit 1, 2 and 3. All boxes will be labeled for study number, center number, subject identifier, prescription instructions, investigator’s name, storage conditions, manufacturing and expiry of dates, and the descriptions of “**Clinical trial use only**” and “**Keep out of reach of children**”. The label on the box will be recored on appropriate page of CRF.

The quantity of capsules in the box for Visit 1, 2 and 3 are packaged as follow: each pack of 100 capsules.

5.2.5 Handling and Storage

The IP will be handled by the investigator or the designated pharmacist for management and dispensation.The IP should be stored at -18±2 ℃, protected from light and humidity.

5.2.6 Product Accountability

All subjects will be given a pre-fixed quantity of IP at Visit 1, 2 and 3. The quantity given will be recorded on the CRF and the drug accountability log. At the time that the un-used drugs are returned by the subject in empty bottles/blisters and box, capsules will be counted at site, and the number left will be recorded on the CRF. Subject should record daily information on the DRC whether the IP is taken in accurate quantity or not.

5.2.7 Assessment of Compliance

Subject's treatment compliance will be assessed by the following formula.

The compliance will be calculated:

$$\frac{\text{Capsule counts actually used}}{\text{Capsule counts prescribed to be used}}$$

Subjects with poor drug compliance, defined as less than 75% mean drug compliance.

5.2.8 Treatment for Investigational Product Overdose

Considering the fact that *L. reuteri* ADR-1 and GMNL-263 has aL. reuterieady been marketed as food supplements, overdose situation will be treated as supportive treatment for subjects. However, the subject or the person caring for the subject should contact the investigator by phone whether any immediate attention is needed in case of a suspected overdose. Regardless of the need for immediately hospital medical care for the overdose event, the subject should be arranged to visit the clinic as soon as possible for evaluation before the investigator continues any further treatment. This post-overdose visit can be considered as an additional visit or as a regular study visit. The monitor appointed by the sponsor should contact the investigator immediately in case of any over-dose event after he/she is aware of the event.

The investigator should consider the following actions for the post-overdose visit:

1. Ask the subject to come back to the clinic and observe the overdosed lesions to see if any further medical treatment is needed for resolving complications caused by overdose of the study medication.
2. Determine whether the subject is suitable for continuing any further treatment or not.
3. Determine whether the subject will be withdrawn from the study due to reasons listed in **Section 4.4** or not.
4. Perform all study procedures required for Visit 5.

Any unfavorable effect caused by overdose must be reported as an AE or SAE.

5.3 Study Assessment

5.3.1 Informed Consent

All subjects must provide signed and dated informed consent form at Visit 0, prior to any study-related procedures. Only the informed consent form approved by institutional review board (IRB) and the applicable health authorities can be used.

5.3.2 Eligibility

Eligibility should be thoroughly checked by the investigator at Visit 0 and 1. See **Section 4.2 and 4.3** for detailed eligibility criteria (inclusion and exclusion criteria).

5.3.3 Height and Body Weight

Height is measured for the subject not wearing shoes at Visit 0, 1, 2, 3 and 4. The measurement of height will be rounded to nearest centimeter.

Body weight is measured for the subject not wearing shoes but wearing light clothing at Visit 0, 1, 2, 3 and 4. The measurement of weight will be rounded to nearest kilogram.

5.3.4 Demography

Demographic data including date of birth and gender will be obtained at Visit 1.

5.3.5 Medical History

The diabetes medical history up to 6 months before entering the study should be recorded at Visit 0. The medical history should include procedural and surgical histories within 1 month.

5.3.6 Urine Pregnancy Test

Subjects with childbearing potential must be confirmed not being pregnant at Visit 0 and must avoid pregnancy during the entire screening and treatment period of this study.

5.3.7 Vital Signs

Blood pressures, pulse rate, respiratory rate and body temperature of subjects will be measured at Visit 0, 1, 2, 3 and 4.

5.3.8 Physical Examinations

Subject will be examined at Visit 0, 1, 2, 3 and 4 by standard physical examination items, including skin, eyes, ENT, cardiovascular system, respiratory system, gastro-intestinal system, blood and blood forming organs, mental status, and other body systems if applicable for describing the status of subject's health.

5.3.9 Hematology Tests

The hematology tests, including white blood cell differential count, white blood cells, red blood cells, hemoglobin, hematocrit, platelet, MCV, MCH, MCHC and RDW will be taken at Visit 0, 2 and 3.

5.3.10 Biochemistry Tests

The biochemistry tests, including AST, ALT, Blood urea nitrogen (BUN), Blood Creatinine, Triglyceride (TG), Cholesterol (CHOL), Low-density cholesterol (LDL-C), High-density lipoprotein cholesterol (HDL-C), LDL-C/ HDL-C, CHO/HDL, Fasting plasma glucose (FPG), Glycated hemoglobin (HbA1c), Insulin, C- peptide, Insulin resistance (HOMA-IR), Free fatty acid (FFA), will be taken at Visit 0, 2 and 3.

5.3.11 Urinalysis Tests

The urinalysis tests, including white blood cells, red blood cells, pH, protein, glucose, and ketone body, Urine micro albumin, Urine creatinine, Glomerular filtration rate (GFR), Urine albumin / creatinine ratio (ACR) will be taken at Visit 0, 2 and 3..

5.3.12 Previous Medications

Previous medications are categorized into diabetes medication and other medication. The diabetes medication should be recorded for at least 6 months preceding the participation of the study (Visit 0). All medical procedures used to treat or prevent diabetes mellitus (DM) should be considered as diabetes medication. Other medications should only be recorded up to 1 month before the subjects enter the study.

5.3.13 Subject Identifier

Subjects will be assigned consecutive numbers as they enter the study. In the study, the screening numbers and randomization numbers are used as subject identifiers. Subject signed the informed consent form will be assigned a sequential screening number in **3** digits with an initial alphabet “S” at screening visit (Visit 0). All enrolled subjects meet the eligible criteria will be randomly assigned to one of the study groups by randomization process. The randomization list will be provided by Bestat Pharmaservics Corp. CRO using SAS to automate the random numbers, which is composed of **3** digits with an initial alphabet “R”.

5.3.14 Inflammatory markers and Cytokines

C-reactive protein (CRP), Interleukin 6 (IL-6), Tumor necrosis factor alpha (TNFα), Interleukin-10 (IL-10), Interleukin-17 (IL-17), Interleukin-1β(IL-1β) concentrations of subjects will be tested before treatment (Visit 0). Same tests will be performed at Visit 3.

5.3.15 Concomitant Medications

All concomitant medications starting from Visit 0 to Visit 4 should be recorded.

5.3.16 Investigational Drug

Investigational drugs will be dispensed to the eligible subjects starting from Visit 1 and followed by Visit 2, and 3. The subjects will be asked to return the unused drugs at Visit 2, 3, and 4. Drug accountability and inventory will be recorded on the case report forms and the relevant study log.

5.3.17 Adverse Event (AE)

The subject will be asked to report adverse event voluntarily and the investigator will also exam the subject for identifying adverse event at each visit during the study, starting from Visit 1.

5.4 Duration of Treatment

The duration of the study for all subjects is approximately 280 days (screening phase for 10 days, treatment phase for 180 days and fllow-up phase for 90 days). The subject will be required to reach a total of 5 visits.

5.5 Concomitant Medication or Treatment

5.5.1 Permitted Treatments

The investigator should try to minimize the concomitant treatments for the subject during the study. If the concomitant treatments are deemed necessary, the investigator should try to maintain stable dosage and therapy type during the study in order to minimize possible interferences for the study endpoint assessments.

5.5.2 Prohibited Treatments

Investigator will try to maintain the dosage of concomitant medications for the subjects during the trial. Subjects are not allowed to take antibiotics and steroids during the study. If need, please recorded on the CRF.

In addition, subjects are not allowed to take any oral systematic antibiotics within the week prior to the primary evaluation at final visit (Visit 3).

6. STUDY MEASUREMENT AND VARIABLES

6.1 Patient Characteristics

Demographic data, including subject’s initials, sex, date of birth, height,

weight, waistline, hip circumference, medical history, and previous medication history, will be recorded.

6.2 Study Procedures

After the beginning of the trial, subjects with DM will be asked to provide their informed consent form and will be screened for enrollment in the study.

6.2.1 Screen Visit

Visit 0：Day -10 day of the first treatment day

- Explain the nature of the study to subjects and have subjects read and sign the Informed Consent Form
- Screen subjects for inclusion and exclusion criteria
- Obtain height, body weight, hieght, waistline and hip circumference
- Obtain demographic characteristics
  - Date of birth
  - Gender
- Obtain diabites mellitus and general medical history
- Perform urine pregnancy test for applicable subjects only
- Obtain vital signs
  - Blood pressures
  - Pulse rate
  - Respiratory rate
  - Body temperature
- Perform physical examinations
  - Skin
  - Eyes
  - Ear, nose, and throat
  - Cardiovascular system
  - Respiratory system
  - Gastro-intestinal system
  - Blood and blood forming organs
  - Mental status
  - Other system

- Take blood samples for C-reactive protein (CRP), Interleukin 6 (IL-6), Tumor necrosis factor alpha (TNFα), Interleukin-10 (IL-10), Interleukin-17 (IL-17), Interleukin-1β (IL-1β) measurements
- Perform hematology tests

- White blood cell differential count	- Platelet
- White blood cells	- MCV
- Red Blood cells	- MCH
- Hemoglobin	- MCHC
- Hematocrit	- RDW

- Perform biochemistry tests

- Aspartate transaminase (SGOT or AST)	- LDL-C/ HDL-C
- Alanine aminotransferase (SGPT or ALT)	- CHO/HDL
- Blood urea nitrogen (BUN)	- Fasting plasma glucose (FPG)
- Blood Creatinine	- Glycated hemoglobin (HbA1c)
- Triglyceride (TG)	- Insulin
- Cholesterol (CHOL)	- C- peptide
- Low-density cholesterol (LDL-C)	- Insulin resistance (HOMA-IR)
-High-density lipoprotein cholesterol (HDL-C)	- Free fatty acid (FFA)

- Perform urinalysis tests

- White blood cells	- Ketone body
- Red blood cells	- Urine micro albumin
- pH	- Urine creatinine
- Protein	- Glomerular filtration rate (GFR)
- Glucose	- Urine albumin / creatinine ratio (ACR)

- Record previous medications (Previous medications should be recorded up to 6 months prior to the first investigational product administration)
- Identifier assigned
- Diet recorded by subject on DRC 7days before next visit
- Dispense DRC to the subject
- Record concomitant medications
- Dispense the box for stool collecting to the subject

6.2.2 Randomization Visit

Visit 1：Month 0, Day 0 (+10~0 day)

- Obtain vital signs
  - Blood pressures
  - Pulse rate
  - Respiratory rate
  - Body temperature
- Inclusion and exclusion criteria confirmation
- Obtain body weight, hieght, waistline and hip circumference
- Assign randomization number to eligible subjects
- Diet recorded by subject on DERC 7days before visit 1
- Dispense new DERC to the subject
- Retrieve the previously given DERC and review DRC by the investigator
- Record concomitant medications
- Dispense investigational product to the subject
- Dispense the box for stool collecting to the subject
- Record adverse event

6.2.3 Evaluation Visits

Visit 2：3rd Month, Day 90 (-10~+0 day)

- Obtain height, body weight, hieght, waistline and hip circumference
- Obtain vital signs
  - Blood pressures
  - Pulse rate
  - Respiratory rate
  - Body temperature
- Take blood samples for C-reactive protein (CRP), Interleukin 6 (IL-6), Tumor necrosis factor alpha (TNFα), Interleukin-10 (IL-10), Interleukin-17 (IL-17), Interleukin-1β (IL-1β) measurements
- Perform hematology tests

- White blood cell differential count	- Platelet
- White blood cells	- MCV
- Red Blood cells	- MCH
- Hemoglobin	- MCHC
- Hematocrit	- RDW

- Perform biochemistry tests

- Aspartate transaminase (SGOT or AST)	- LDL-C/ HDL-C
- Alanine aminotransferase (SGPT or ALT)	- CHO/HDL
- Blood urea nitrogen (BUN)	- Fasting plasma glucose (FPG)
- Blood Creatinine	- Glycated hemoglobin (HbA1c)
- Triglyceride (TG)	- Insulin
- Cholesterol (CHOL)	- C- peptide
- Low-density cholesterol (LDL-C)	- Insulin resistance (HOMA-IR)
-High-density lipoprotein cholesterol (HDL-C)	- Free fatty acid (FFA)

- Perform urinalysis tests

- White blood cells	- Ketone body
- Red blood cells	- Urine micro albumin
- pH	- Urine creatinine
- Protein	- Glomerular filtration rate (GFR)
- Glucose	- Urine albumin / creatinine ratio (ACR)

- Diet and exercise recorded by subject on DERC 7days before visit 2
- Dispense new DERC to the subjects
- Retrieve the previously given DERC and review DERC by the investigator
- Retrieve and Dispense the box for stool collecting to the subject
- Record concomitant medications
- Dispense new investigational product to the subject
- Retrieve unused investigational product
- Record adverse event

Visit 3：6th Month, Day 180 (-10~+7 day)

- Obtain height, body weight, hieght, waistline and hip circumference
- Obtain vital signs
  - Blood pressures
  - Pulse rate
  - Respiratory rate
  - Body temperature
- Perform physical examinations
  - Skin

- Eyes
- Ear, nose, and throat
- Cardiovascular system
- Respiratory system
- Gastro-intestinal system
- Blood and blood forming organs
- Mental status
- Other system

➢ Take blood samples for C-reactive protein (CRP), Interleukin 6 (IL-6), Tumor necrosis factor alpha (TNFα), Interleukin-10 (IL-10), Interleukin-17 (IL-17), Interleukin-1β (IL-1β) measurements

➢ Perform hematology tests

- White blood cell differential count	- Platelet
- White blood cells	- MCV
- Red Blood cells	- MCH
- Hemoglobin	- MCHC
- Hematocrit	- RDW

➢ Perform biochemistry tests

- Aspartate transaminase (SGOT or AST)	- LDL-C/ HDL-C
- Alanine aminotransferase (SGPT or ALT)	- CHO/HDL
- Blood urea nitrogen (BUN)	- Fasting plasma glucose (FPG)
- Blood Creatinine	- Glycated hemoglobin (HbA1c)
- Triglyceride (TG)	- Insulin
- Cholesterol (CHOL)	- C- peptide
- Low-density cholesterol (LDL-C)	- Insulin resistance (HOMA-IR)
-High-density lipoprotein cholesterol (HDL-C)	- Free fatty acid (FFA)

➢ Perform urinalysis tests

- White blood cells	- Ketone body
- Red blood cells	- Urine micro albumin
- pH	- Urine creatinine
- Protein	- Glomerular filtration rate (GFR)
- Glucose	- Urine albumin / creatinine ratio (ACR)

➢ Diet and exercise recorded by subject on DERC 7days before visit 2

➢ Dispense new DERC to the subjects

- Retrieve the previously given DERC and review DERC by the investigator
- Retrieve the box for stool collecting to the subject
- Record concomitant medications
- Dispense new investigational product to the subject
- Retrieve unused investigational product
- Record adverse event

6.2.4 Final Visit

Visit 4：9th Month, Day 270 10 day

- Obtain height, body weight, hieght, waistline and hip circumference
- Obtain vital signs
  - Blood pressures
  - Pulse rate
  - Respiratory rate
  - Body temperature
- Perform physical examinations
  - Skin
  - Eyes
  - Ear, nose, and throat
  - Cardiovascular system
  - Respiratory system
  - Gastro-intestinal system
  - Blood and blood forming organs
  - Mental status
  - Other system
- Perform biochemistry tests
  - Glycated hemoglobin (HbA1c)
- Record concomitant medications
- Dispense new investigational product to the subject
- Retrieve unused investigational product
- Record adverse event
- Complete exit form

6.3 Clinical Efficacy Assessments

Net changes in blood sugar and lipid profile based on subjects will be the

primary efficacy variable.

6.4 Clinical Safety Assessments

Safety profile will be evaluated by the following line-up of endpoints:

1. Adverse events
2. Vital signs
3. Changes in physical examinations
4. Net changes from pre-treatment in hematology or biochemistry tests results
5. Changes in urinalysis tests

7. DISCONTINUATION OF SUBJECTS

Subjects will be discontinued from the study trial if any of the following occur:

1. Subjects withdraw consent
2. At the specific request of the Safety Data Review Board
3. An intercurrent illness affects assessment of clinical status to a significant degree at the investigator’s discretion
4. Poor compliance with protocol
5. Subject is pregnant
6. Investigator concludes to discontinue the study in subject’s best benefit

The date and reason should be identified and recorded on subject’s Case Report Form (CRF). Reasonable effort should be made to complete the appropriate assessments. Subject will be considered **off study** when withdrawal or death occurs. The investigator must notify the Sponsor of the discontinuation of a subject at once when a serious or unexpected AE associated with the use of the study medication occurs.

8. Adverse Events (AE) and Serious Adverse Events (SAE)

8.1 Definition of AE

An adverse event is defined as any untoward medical occurrence in a subject or clinical investigation subject administered a pharmaceutical product and which does not necessarily have a causal relationship with this treatment. An adverse event can therefore be any unfavorable and unintended sign (including an abnormal laboratory finding), symptom, or disease temporally associated with the use of a medicinal (investigational) product, whether it is considered as relating to the medicinal (investigational) product or not.

Pre-existing conditions will be recorded as baseline on the Medical History of CRF. If the pre-existing condition does not change, it does not have to be reported as an AE on subsequent cycles.

All clinical AEs encountered during the study period will be reported on an AE page of the CRF.

The severity of AEs will be graded as a five-point scale and reported on the CRF.

Mild	Transient or slight discomfort is noticed but no medical intervention or therapy is needed
Moderate	Discomfort is sufficient to reduce or affects daily activity
Severe	Unable to work or perform normal daily activity
Life-threatening	Represents an immediate threat to life
Death	Death

AEs will be graded and reported. All AEs, regardless of severity, will be followed by the investigator until resolution is satisfactory. The relationship of each event to treatment will be assessed by the investigator and recorded on the CRF.

8.2 Definition of SAE

A serious adverse event is defined as any untoward medical occurrence, whether or not considered treatment-related, that might be happen at any dose and:

- results in death
- is life-threatening (defined as an event in which the subject is at immediate risk of death at the time of the event; Life-threatening does not refer to an event that hypothetically might have caused death if it were more severe, resulting in permanent or significant disability/incapacity)
- requires inpatient hospitalization or prolongation of existing inpatient hospitalization
- results in persistent or significant disability/incapacity
- leads to a congenital anomaly or birth defect
- events requiring medical and/or surgical intervention to prevent permanent impairment of function or permanent damage to a body structure

All serious adverse events will be reported on Serious Adverse Event forms, which will be completed by the investigator. A copy of the Serious Adverse Event form will be sent to the sponsor or its delegate and will be filed in the site files.

All non-serious adverse events, regardless of its severity, whether or not it is ascribed to the study, should be recorded in the CRF. All SAEs must be reported immediately to the Sponsor. All SAEs will be reported by facsimile upon becoming aware of the event within 24 hours to the sponsor or sponsor's delegate. The Investigator must inform SAE to local IRB. The sponsor or the sponsor's delegate will notify health authority of the fatal and life-threatening SAE in 7 calendar days, followed by a report within additional 8 calendar days. Other SAEs must be filed as soon as possible but no later than 15 calendar days. The copy of each report will be kept in investigator's files.

8.3 Clinical Laboratory Abnormalities

Abnormal laboratory findings and/or assessments that are judged by the investigator as clinically significant will be reported as AEs or SAEs if they meet the definitions of set forth.For the condition that the abnormal laboratory and/or assessment findings are considered as the underlying disease related, and are not unexpectedly worsened during the study, no AE or SAE will be reported.

8.4 Reporting and Recording of AE and SAE

All AEs and SAEs must be documented in the source documents and the relevant

CRF and SAE form when applicable. The investigator may be asked to provide photocopies of the medical records for completion of AE or SAE report. The subjects' names on the medical records submitted to the relevant parties will be concealed. It is the investigator's responsibility to report AEs or SAEs by diagnosis terminologies, if possible. When the diagnosis is possible for the reported AE or SAE, no signs and symptoms used to establish that particular diagnosis should be reported.

The investigator will be asked to determine the intensity and causality of each AE and SAE based on the investigator's clinical judgment. Three levels of intensity and five levels of causality are used to evaluate each AE and SAE as shown below:

Intensity

- Mild: Easily tolerated by the subject, causing minimal discomfort and not interfering with daily activities.
- Moderate: Sufficiently discomforting and interfering with normal daily activities.
- Severe: Make normal daily activities prevented.

*There is no direct relationship between severity and seriousness of an AE.

Causality

- Highly probable: Should be reserved for those events which have no related uncertainty in their relationship to test drug administration.
- Possible: The suspected adverse event may follow a reasonable temporal sequence from test drug administration but could have been produced or mimicked by the patient's clinical state or by other modes of therapy concomitantly administered to the subject.
- Unlikely: Whether there has been a temporal relationship between the test drug and the suspected adverse event or nor, it is more likely than not that the event could have been produced by the patient's clinical state or other modes of therapy administered to the patient.
- Unrelated: Should be reserved for those events which occur prior to unrelated test drug administration (i.e., washout or single-masked placebo) or those events which cannot be even remotely related to study participation (e.g., injuries sustained in an automobile accident).

It is usually important for the investigator to take information of underlying diseases, concomitant drugs, temporal relationship of the onset of the event to the time of dosing the IP, and re-challenging outcomes, into account when the investigator is making a causal relation decision.

It is investigator's responsibility to proactively follow the outcome of each AE/SAE until resolution or stabilization of the condition or lost of follow-up. Serious, alarming and/or unusual adverse events must be reported to one of the following individuals within 24 hours of the investigator's knowledge of the event:

POSITION	NAME	AFFILIATION	TELEPHONE NUMBER	FAX NUMBER
SAE Contact	Ya-Hui Chen	GenMont Biotech. Inc.	+886-6-505-2151 ext 326	+886-6-505-5152

An Adverse Event Form should be completed for all serious adverse events and forwarded to the sponsor within 24 hours. When new significant information is obtained as well as when outcome of an event is known, the investigator should inform the sponsor. In applicable cases, sponsor may request a letter from the investigator summarizing events related to the case. Investigators should follow subjects as far as possible until an outcome to the events is known.

The investigator is responsible to communicate details of medical emergencies in trial patients to the Ethics Committee. Sponsor is responsible to inform the events to the regulatory authorities.

9. DATA MANAGEMENT AND EVALUATION

9.1 Data Management

A paper CRF is the method for recording data. All entries on the CRF are made in English and are supported by the source data. All data management procedures are written in a separate document. The database from the CRFs will be set up in excel file. A comparison of the double data entry files will be conducted, and discrepancies between the two data entry personnel will be

resolved by consulting the original CRF. All data will send to analysis by Best At Pharmaservices Corp.

9.2 Sample Size

In this study, the difference between the two study groups is derived as 1.0 from phase II study result and the assumed standard deviation (SD) is 2.5. Using two-sample t-test at a 0.025 one-sided significance level, total sample size of 120 subjects will achieve statistical power of 80%.

As a dropout rate of 25% is considered, approximately 120 subjects are planned to be enrolled into this study to complete a total of 90 evaluable subjects.

9.3 Analyzed Population

Data analyses and summaries of efficacy and safety assessment will be performed in the following populations:

Intent-to-treat (ITT) population - all randomized subjects who take at least one dose of study medication and have at least one follow-up primary efficacy measurement regardless of their compliance with the protocol.

Per-protocol (PP) population - it is a subset of ITT population and all randomized subjects included in this population should satisfy the following conditions:

1. Take at least 75% of total study medications
2. With primary efficacy measurement
3. Without taking any prohibited medications

Safety population - all randomized subjects who have taken at least one dose of study medication and completed the protocol.

Efficacy analysis and safety population will be performed on PP populations.

9.4 Statistical and Analytical Methods

9.4.1 Statistical Analysis of Demographic and Baseline Data

Categorical variables (for example, gender) will be tabulated by frequencies and percentages. For the comparability between two study groups, Chi-square test will be used as the statistical methodology, or Fisher's exact test will be adopted if normal approximation to the binomial distribution is violated. Continuous variables (for example, age, height and weight) will be summarized by the number of observation, mean, median, standard deviation, minimum, maximum, and 95% confidence interval. If statistical test is conducted, T test will be used or Wilcoxon rank sum test will be adopted when normal assumption is violated. The statistical tests will be conducted under a two-tailed, significance level of 0.05 with related p-value.

All efficacy analysis will be adjusted for the demographic characteristic which is statistical significance between three study groups at baseline.

9.4.2 Statistical Analysis of Efficacy Endpoints

For each efficacy evaluation based on subject's change of blood sugar and lipid profiles.

Primary endpoint

The change level between baseline and 6th month treatment of blood sugar and blood lipid will be analyzed to assess the effect of daily intake of L. reuteri ADR-1 and ADR-3 on type 2 DM subjects treatment.

The primary analysis to test the difference of the net change in blood sugar and blood lipid between three study groups will be conducted using T test with adjustment for statistical significant baseline characteristics. Wilcoxon rank-sum test will be used if the data with the violation of the normal assumption. The statistical tests for primary efficacy endpoint will be conducted based on a one-tailed with significance level of 0.025.

Secondary endpoint

For secondary efficacy endpoints such as:

- Net changes in insulin resistance related markers rated by subjects at the 6th month evaluations from baseline

- Net changes in inflammatory makers and cytokines rated by subjects at the 6th month evaluations from baseline
- Net changes in antioxidant index rated by subjects at the 6th month evaluations from baseline
- Division in gut microbiota profile rated by subjects at the 6th month evaluations from baseline

Comparisons of the results change between the probiotic and placebo groups were analyzed using the Two sample t-test. Correlation between the variables was computed by Spearman's rho correlation provided by PASW Statistics 18 Software (SPSS Inc).

9.4.3 Statistical Analysis of Safety Endpoints

Adverse events (AE)

In analysis of adverse events, the Medical Dictionary for Regulatory Activities (MedDRA) adverse event dictionary will be used to map verbatim adverse events to preferred terms and system organ class. The tabulations will count the number of subjects reporting individual adverse events. In addition, the incidence of adverse events will be categorized by severity and be related to the time of occurrence.

Changes in physical examinations

Frequency tables for physical examination by study groups will be provided. Listings of subjects with abnormal findings during the study will also be provided.

Safety assessment in hematology and biochemistry analysis

The listing of subject with abnormal values and significant changes in laboratory examination (hematology and biochemistry tests) will be presented. Net change from pre-treatment in laboratory test results will be summarized and analyzed by descriptive statistics.

Changes in urinalysis tests

The urinalysis assessment results for categorical data type will be tabulated with related numbers and percentages. Assessments for continuous data

type will be calculated with mean and standard deviation by each group.

Vital signs

The vital signs, including blood pressures, pulse rate, respiratory rate and body temperature, will be summarized by descriptive statistics at baseline (screening visit), final visit and the mean change from baseline. Paired T-test will be used for evaluation of the change from baseline within study group. To compare the change between the two study groups from baseline, T-test will be used or Wilcoxon rank-sum test will be adopted if the normal assumption is violated.

9.5 Premature Termination and Missing Values

The dropouts, premature termination of study medication, and withdrawal will be summarized and the dates and reasons for terminations will be provided.

For primary endpoint, the last-observation-carried-forward (LOCF) procedure will be applied to estimate the missing data. No imputation will be done for estimating the missing value for safety variables.

10. ETHICAL AND LEGAL ASPECTS

10.1 Local Regulations / Declaration of Helsinki

The principles of the “Declaration of Helsinki” or the laws and regulations of the country in which the research is conducted must be followed in all study procedures, conduction, evaluation, and documentation throughout the study. The study must fully adhere to the principles outlined in “Guideline for Good Clinical Practice” ICH Tripartite Guideline or with local law if it affords greater protection to the patient.

10.2 Independent Ethics Committees / Institutional Review Board

Before the enrollment of subjects, the study must be approved by the Institutional Review Board (IRB). This protocol and any accompanying material provided to the patient (such as patient information sheets or descriptions of the study used to obtain informed consent) as well as any

advertising or compensation given to the patient, will be submitted by the investigator to an Independent Ethics Committee. The comments of the IRB must be dated and filed. After receiving the approval letter from the IRB, the investigators have the responsibility to forward the copy of the approval letter to GENMONT BIOTECH INC. before the commencement of the clinical trial.

During the trial, the Investigator is required to send various documents to the IRB for review:

1. Changes to the current protocol
2. All protocol amendments and Patient Informed Consent Form revisions
3. Reports of AEs that are serious, unexpected, and associated with the investigational drug, and any life-threatening problems, or death

10.3 Subject Information and Informed Consent

The informed consent document should meet the requirements of the latest version of the Declaration of Helsinki and any applicable regulations and guidelines. It must be approved by an IRB.

It is the responsibility of the investigator, or a person designated by the investigator, to obtain written informed consent from each subject participating in this study after adequate explanation of the aims, methods, objectives and potential hazards of the study. It must also be explained to the subjects that they are completely free to refuse to enter the study or to withdraw from it at any time for any reason. Each patient will be told that his or her records may be accessed by authorized personnel of the site and other authorized individuals without violating the subject’s confidentiality, to the extent permitted by the applicable laws and/or regulations. Form must be obtained from each patient or his or her legal representative; a copy will be given to the person signing the form.

If any modifications are to be made according to local requirements, the revised version must be approved by GENMONT BIOTECH INC. and IRB.

10.4 Subject Data Protection

All patients will be identified by the subject number, initials, date of birth and gender in the CRFs. Subject’s name will not be supplied to the sponsor. The investigator must assure that subjects' anonymity will be maintained and that

their identities are protected from unauthorized parties. The subjects should be informed about the possibility of audits by authorized representatives of the company and regulatory authorities when audit or review is required.

10.5 Protocol Amendments

Any change of this protocol that affect study objectives, study design, study procedures, patient population, or significant administrative procedures will require a formal amendment to the protocol. Any proposed protocol amendments must be sent in written form to the applicable IRB. Prior to implementation, an amendment must be approved by the Sponsor, the Investigator and the applicable IRB.

11. STUDY DOCUMENTATION

11.1 Source Documentation and Records Retention

A file for each subject must be maintained that includes the signed informed consent form and copies of all source documentation related to that subject. For each subject treated with the study drug(s), the Principal Investigator is required to prepare and maintain case histories that include all observations and other data pertinent to the investigation. This will include all source documents needed to verify the accuracy of all observations and other data contained in the CRFs on each study patient.

The Investigator or his/her designee is required to retain the records related to the trial for a period of 2 years following the date of marketing application is approved for the indication of being investigated. If no application is to be filed or if the application is not approved for such indication, the records must still be retained until 2 years after the investigation is discontinued and the regulatory agencies are notified.

The Investigator shall retain study drug disposition records and source documents for the maximum period required by the country and the institution in which the study has been conducted, or for the period specified by the Sponsor, whichever is longer. The Investigator must contact the Sponsor prior to destroying any records associated with the study.

11.2 Source Documents and Background Data

The investigator shall supply the sponsor on request with any required background data from the study documentation or clinic records. This is particularly important when Case Report Forms are illegible or when errors in data transcription are suspected. In case of special problems and/or governmental queries or requests for audit inspections, it is also necessary to have access to the complete study records, providing protection of subject's confidentiality.

11.3 Case Report Form (CRF)

CRFs will be supplied for recording all data from each subject. CRFs must be typewritten or printed legibly using black ballpoint pen or completed electronically. The investigator or his/her designee is responsible for recording all data relating to the trial on the CRFs. The investigator must verify that all data entries on the CRFs are accurate and correct by signing and dating the CRF on the designated pages.

If an item is not available or is not applicable, it should be documented as such; no blank spaces should be left on a CRF.
For subjects removed from study, the Sponsor must be notified by e-mail within 24 working hours of the removal.

12. REFERENCES

Yun SI, Park HO and Kang JH. Effect of Lactobacillus gasseri BNR17 on blood glucose levels and body weight in a mouse model of type 2 diabetes, J. Appl. Microbiol.,107:1681-1686 (2009).

Yadav H, Jain S, Sinha, PR. Oral administration of dahi containing probiotic Lactobacillus acidophilus and Lactobacillus casei delayed the progression of streptozotocin-induced diabetes in rats, J. Dairy Res.75:189-195 (2008).

Membrez, M, Blancher, F, Jaquet, M, Bibiloni, R, Cani, PD, Burcelin, RG, Corthesy, I, Macé, K and Chou, CJ. Gut microbiota modulation with norfloxacin and ampicillin enhances glucose tolerance in mice. FASEB J. 22:2416-2426 (2008).

Larsen N, Vogensen FK, van den Berg FW, Nielsen DS, Andreasen AS, Pedersen BK, Al-Soud WA, Sørensen SJ, Hansen LH and Jakobsen M. Gut Microbiota in Human Adults with Type 2 Diabetes Differs from Non-Diabetic Adults. PLoS ONE. 5: e9085 (2010).

Lee HB, Yu MR, Yang Y, Jiang Z, Ha, H. Reactive oxygen speciesregulated signaling pathways in diabetic nephropathy. J. Am. Soc. Nephrol.14:S241-S245 (2003).

Ha H and Lee HB. Reactive oxygen species and matrix remodeling in diabetic kidney, J. Am. Soc. Nephrol. 14:S246-S249 (2003).

Ejtahed HS, Mohtadi-Nia J, Homayouni-Rad A, Niafar M, Asghari-Jafarabadi M and Mofid V. Probiotic yogurt improves antioxidant status in type 2 diabetic patients Nutrition. 28:539-43 (2012).

Mazloom Z, Yousefinejad A and Dabbaghmanesh MH. Effect of probiotics on lipid profile, glycemic control, insulin action, oxidative stress, and inflammatory markers in patients with type 2 diabetes: a clinical trial. Iran J Med Sci. 38(1):38-43 (2013).

Asemi Z, Samimi M, Tabassi Z, Naghibi Rad M, Rahimi Foroushani A, Khorammian H and Esmaillzadeh A. Effect of daily consumption of probiotic yoghurt on insulin resistance in pregnant women: a randomized controlled trial. Eur J Clin Nutr. 67:71-4 (2013).

Kung WJ, Lin CC, Liu SH, Chaung HC. Association of interleukin-10 polymorphisms with cytokines in type 2 diabetic nephropathy. Diabetes Technol Ther. 12:809-13 (2010).

Ejtahed HS, Mohtadi-Nia J, Homayouni-Rad A, Niafar M, Asghari-Jafarabadi M, Mofid V, Akbarian-Moghari. Effect of probiotic yogurt containing Lactobacillus acidophilus and Bifidobacterium lactis on lipid profile in individuals with type 2 diabetes mellitus. A. J. Dairy Sci. 94:3288–3294 (2011).

Moroti C, Souza Magri LF, de Rezende Costa M, Cavallini DC, Sivieri K. Effect of the consumption of a new symbiotic shake on glycemia and cholesterol levels in elderly

people with type 2 diabetes mellitus. Lipids Health Dis. 11:29 (2012).

Hsieh FC, Lee CL, Chai CY, Chen WT, Lu YC, Wu CS. Oral administration of Lactobacillus reuteri GMNL-263 improves insulin resistance and ameliorates hepatic steatosis in high fructose-fed rats. Nutr Metab (Lond).10:35 (2013).

Lu YC, Yin LT, Chang WT, Huang JS. Effect of Lactobacillus reuteri GMNL-263 treatment on renal fibrosis in diabetic rats. J Biosci Bioeng. 110:709-15 (2010).
